# Association of soft drinks and 100% fruit juice consumption with risk of cancer: a systematic review and dose–response meta-analysis of prospective cohort studies

**DOI:** 10.1186/s12966-023-01459-5

**Published:** 2023-05-15

**Authors:** Bei Pan, Honghao Lai, Ning Ma, Dan Li, Xiyuan Deng, Xiaoman Wang, Qian Zhang, Qiuyu Yang, Qi Wang, Hongfei Zhu, Mengting Li, Xiao Cao, Jinhui Tian, Long Ge, Kehu Yang

**Affiliations:** 1 Evidence-Based Medicine Center, School of Basic Medical Sciences, Lanzhou University, No. 199 Donggang West Road, Chengguan District, Lanzhou, 730000 China; 2 Evidence-Based Social Science Research Center, School of Public Health, Lanzhou University, No. 199 Donggang West Road, Chengguan District, Lanzhou, 730000 China; 3 Key Laboratory of Evidence Based Medicine and Knowledge Translation of Gansu Province, Lanzhou, 730000 China; 4 Gansu Provincial Maternity and Child-care Hospital , Lanzhou, 730000 China; 5 Evidence-Based Nursing Center, School of Nursing, Lanzhou University, Lanzhou, 730000 China

**Keywords:** Sugar-sweetened beverages, Artificially sweetened beverages, Fruit juices, Cancer, Dose–response meta-analysis

## Abstract

**Background:**

Studies of the associations between soft drinks and the risk of cancer showed inconsistent results. No previous published systematic reviews and meta-analysis has investigated a dose–response association between exposure dose and cancer risk or assessed the certainty of currently available evidence. Therefore, we aim to demonstrate the associations and assessed the certainty of the evidence to show our confidence in the associations.

**Methods:**

We searched Embase, PubMed, Web of Science, and the Cochrane Library from inception to Jun 2022, to include relevant prospective cohort studies. We used a restricted cubic spline model to conduct a dose–response meta-analysis and calculated the absolute effect estimates to present the results. The Grading of Recommendations Assessment, Development and Evaluation (GRADE) approach was used to assess the certainty of the evidence.

**Results:**

Forty-two articles including on 37 cohorts enrolled 4,518,547 participants were included. With low certainty evidence, increased consumption of sugar-sweetened beverages (SSBs) per 250 mL/day was significantly associated with a 17% greater risk of breast cancer, a 10% greater risk of colorectal cancer, a 30% greater risk of biliary tract cancer, and a 10% greater risk of prostate cancer; increased consumption of artificially sweetened beverages (ASBs)re per 250 mL/day was significantly associated with a 16% greater risk of leukemia; increased consumption of 100% fruit juice per 250 mL/day was significantly associated with a 31% greater risk of overall cancer, 22% greater risk of melanoma, 2% greater risk of squamous cell carcinoma, and 29% greater risk of thyroid cancer. The associations with other specific cancer were no significant. We found linear dose–response associations between consumption of SSBs and the risk of breast and kidney cancer, and between consumption of ASBs and 100% fruit juices and the risk of pancreatic cancer.

**Conclusions:**

An increment in consumption of SSBs of 250 mL/day was positively associated with increased risk of breast, colorectal, and biliary tract cancer. Fruit juices consumption was also positively associated with the risk of overall cancer, thyroid cancer, and melanoma. The magnitude of absolute effects, however, was small and mainly based on low or very low certainty of evidence. The association of ASBs consumption with specific cancer risk was uncertain.

**Trial registration:**

PROSPERO: CRD42020152223

**Supplementary Information:**

The online version contains supplementary material available at 10.1186/s12966-023-01459-5.

## Background

The adverse health effects of artificially sweetened beverages (ASBs), sugar-sweetened beverages (SSBs), and 100% fruit juice have received widespread attention from public and scientific communities [1]. SSBs are the largest source of sugar in the diet, and according to the Global Burden of Disease study, from 1990 to 2016 the summary exposure value of SSBs increased by more than 40% [2]. SSBs intake has been documented to contribute the most to the increased diabetes mortality, cardiovascular disease (CVD) mortality, and disability-adjusted life years (DALYs) [2–4]; It also leads to higher risks of type 2 diabetes [5], hypertension [6], CVD [7], and obesity [8]. Considering the important impact on health, alternative sweeteners are added to soft drinks and labelled as “no added sugar” [9], similar positive association has also been reported between ASB intake and hypertension [10], obesity [8], and type 2 diabetes [1]. Additionally, although 100% fruit juices are perceived as healthier alternative to SSBs because they are rich in bioactive compounds and various nutrients [9], they are also associated with negative health effects may partly owing to less dietary fiber than the whole fruit, additional sugar from juice, and the more amount of energy consumed from juice than whole fruit [11, 12].

Although the associations between SSBs, ASBs, or 100% fruit juice intake and other conditions such as type 2 diabetes, obesity, and cardiovascular disease have been well-documented [1, 7, 8], their association with specific cancer risk remains inconsistent. For example, studies from NutriNet-Santé reported that both SSBs and 100% fruit juice intake were positively associated with the risk of cancer [13], however, these findings were not reflected in the Singapore Chinese Health Study [14]. Several studies [13, 15–17] have also showed that there was no significant association between consumption of ASBs intake and cancer risk except non-Hodgkin lymphoma [18]. Although systematic reviews have been conducted to assess the associations between SSBs, ASBs, or 100% fruit juice intake and different types of cancer, most of them only focused on certain types of cancer such as pancreatic and colorectal cancer, and failed to assess other types of cancer such as overall cancer and breast cancer [13–17]. In addition, published systematic reviews failed to conduct a dose–response meta-analysis to observe the dose-specific association between sweetened beverage exposure dose and cancer risk, or assess the certainty of currently available evidence [19].

This systematic review and dose–response meta-analysis aimed to comprehensively assess the associations between soft drink, SSBs, ASBs, and 100% fruit juice intake and the risk of specific cancers. We rated the certainty (quality) of evidence and interpreted our findings using Grading of Recommendations, Assessment, Development and Evaluation (GRADE) approach [20].

## Methods

We followed the Preferred Reporting Items for Systematic Reviews and Meta-Analyses (PRISMA) checklist [21] to report this systematic review. The protocol for this systematic review was registered in the International Prospective Register of Systematic Reviews (PROSPERO) (CRD42020152223).

### Data sources and searches

We searched Embase, PubMed, Web of Science, and the Cochrane Library from their inception to Jun 20^th^, 2022. Search terms included ‘beverages AND cancer AND cohort’ Detailed search strategies are provided in Supplementary Table S1. We also reviewed the references of previously published systematic reviews and meta-analysis to identified additional potential studies. There were no restrictions on the publication date, language or status of publication.

### Study selection

We included prospective cohort studies with participants aged 18 years or older that reported the most adequately adjusted effect estimates (relative risk (RR), hazard ratio (HR), or odds ratio (OR)) with 95% confidence intervals (CIs). Cohort studies that investigate the associations between SSBs (beverages with added sugar such as corn sweetener, dextrose, glucose, brown sugar, maltose, corn syrup, fructose, raw sugar, honey, lactose, sucrose, molasses, and malt syrup), ASBs (beverages with caffeinated, caffeine-free, and noncarbonated low-calorie diet), or 100% fruit juice (studies that reported 100% fruit juice or fruit juice assessed separately from soft drinks) intake, and the risks of overall cancer or a specific cancer were included. We also included abstracts if the results of the multivariate analysis were reported. We excluded studies that involved patients with any type of cancer at baseline; that were cross sectional and case–control studies; and that included more than 20% of participants with chronic illness at the baseline. We only included data with the latest publication or longest follow-up and most informative with more relevant data on exposure and outcomes, when multiple studies from the same cohort and with same outcomes. There was no upper limit on the age of participants or restriction to publication status. Detailed definitions of exposure are presented in Supplementary Table S2.

We used EndNote X9 to manage the initial searched records; after removing duplicate records, the remaining records were imported to Rayyan, an online literature management platform [22], for titles and abstracts screening. Potential studies were subjected to full-text screening. Using a pre-designed eligibility form (Supplementary Table S3), teams of two reviewers (XC and QZ, QW and HFZ, QW and YQY, MTL and HHL) independently performed the study screening. Disagreements were resolved by consensus.

### Data extraction and quality assessment

We performed two-rounds of calibration exercises using a pre-designed standard data collection form to ensure agreement among the reviewers. Subsequently, two reviewers (MTL and HHL) extracted the data and assessed the risk of bias in duplication. Disagreements were resolved by consensus. The data of interest included the name of the cohort, year of publication, country where cohort was conducted, duration of follow-up, sample size at baseline, age, sex, number of participants within each exposure category, person-years, number of events, effect estimates, and 95% CIs.

To assess the risk of bias of included individual cohort, we used a modified version of the Newcastle–Ottawa Scale that include seven questions [23, 24]. We assessed the risk of bias for each question as “definitely or probably yes” (low risk of bias) or “definitely or probably no” (high risk of bias). We classified each study as having a low risk of bias if five or more of the seven questions were at low risk; otherwise, we classified the studies as having a high risk of bias.

### Certainty of evidence

We used GRADE approach to assess the certainty of evidence for each cancer outcome and categorised evidence certainty as high, moderate, low, or very low [20]. According to the GRADE standard, the certainty of evidence of cohort studies may start at low certainty and could be upgraded to moderate or high certainty if they present a dose–response gradient, a large effect, or if confounders likely minimise the effect [25]. However, evidence certainty could also be downgraded because of serious limitations of the study, including indirectness, inconsistency, publication bias, or imprecision [25].

To calculate the absolute effect, we used the population risk from the Global cancer statistics (GLOBOCAN) as the baseline risk for each specific cancer [26]. We calculated the absolute risk difference by multiplying the pooled effect estimate from the meta-analysis by baseline risk for cancer incidence. When a significant subgroup effect of risk of bias was presented, we only used the results from studies with low risk of bias.

### Data synthesis

We conducted a dose–response analysis as suggested by Greenland and Orsini as the primary analysis [27, 28]. We calculated study-specific pooled RR estimates with corresponding 95% CI for the effect of per 250 mL*/day* increase in SSBs, ASBs, 100% fruit juices, or total soft drink intake. For studies reporting effect estimates as HRs, we assumed that the HRs were approximately equal to the RRs [29]. For analyses that include more than 3 studies, we tested nonlinearity by employing a restricted cubic spline model with knots at the 25th, 50th, and 75th percentiles [27, 28]. The quantity of beverage intake, number of events or person-year, effects estimate with corresponding 95%CI of all exposure categories or other sufficient information to calculate the above details were needed to perform a dose–response meta-analysis. If studies reported the exposure of SSBs, ASBs, 100% fruit juices, and total soft drinks using different unit (such as cups or servings), after we confirmed the most frequently used unit in the included studies (mL/day) and the median volume of beverages (median = 250 mL/day), we standardised measures of the association to ml per day of soft drink consumption.

We compared the highest category with the lowest category of exposure by performing a random effect meta-analysis as a sensitivity analysis.

Statistical heterogeneity among the included studies was examined using Cochrane’s Q test and quantified using the *I*^*2*^ statistic [29]. When 10 or more studies were available, we assessed the publication bias using Begg's rank correlation test with a funnel plot [30]. For the dose–response analysis, we tested subgroup analysis based on age, follow-up duration, sex, study location, and risk of bias. We used meta-regression to calculate the *P* values of the interaction test (P interaction). *P *_*interaction*_ ≤ 0.05 was considered to have statistically significant subgroup difference. We also conducted sensitivity analysis by excluding studies reported SSBs that including 100% fruit juices. Review Manager 5.4 (Nordic Cochrane Center, Copenhagen) and Stata 15.1 (Stata Corp, College Station, TX) were used for all statistical analyses.

## Results

### Study selection

**Fig. 1:**
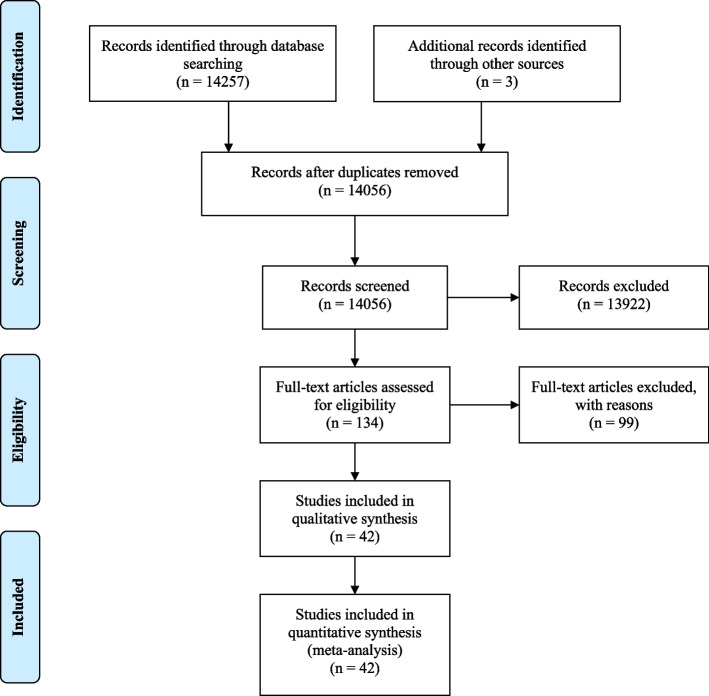
Evidence search and selection

Our search yielded 142,60 records, after screening of titles and abstracts, 134 full-texts were reviewed for eligibility. Finally, 42 articles including on 37 cohorts with 4,518,547 participants were eligible (Fig. 1). Of them, we included 35 studies for dose–response meta-analysis. The list of excluded studies during full-text screening is presented in Appendix 1. A list of included studies is presented in Appendix 2.

### Study characteristics

The baseline characteristics are showed in Table 1 and more details are showed in Supplementary Table S4. In total, 42 articles reported 22 types of cancer and in which 11 types of cancer included more than 2 articles. Seven articles with 416,054 participants reported on breast cancer; 10 articles with 547,713 participants reported on colorectal cancer; 3 articles with 520,114 participants reported on gastric cancer; 8 articles with 1,594,301 participants reported on pancreatic cancer; 6 articles with 209,665 participants reported on prostate cancer; 4 articles with 954,507 participants reported on kidney cancer; 3 articles with 77,229 participants reported on endometrial cancer; 2 articles with 225,470 participants reported on non-Hodgkin lymphoma; and 1 articles reported on esophagus cancer, biliary tract cancer, hepatocellular carcinoma, pharynx cancer, larynx cancer, melanoma, squamous cell carcinoma, thyroid cancer, urothelial cell carcinomas, oral cancer, and ovary cancer, respectively. Articles enrolled participants with a median proportion of women of 59.65% and mean age of 48.05 years. The median follow-up period was 14.00 years.

**Table 1:** Baseline characteristics of included studies

Author, Year	Name of cohort	Country	Sample size	Duration of follow-up (year)	Age, year	Female, %	Types of exposure	Types of outcomes
MICHAUD [31]	The Health Professionals Follow-up Study	USA	47,909	10	NR	0	Fruit juice	Bladder cancer
Ellison [32]	NCS	Canada	3400	NR	50 to 84 years	0	SSB	Prostate cancer
Schernhammer [16]	NHS	USA	88,794	20	46.30	100	SSB; ASB	Pancreatic cancer
Schernhammer [16]	HPFS	USA	49,364	20	47.73	0	SSB; ASB	Pancreatic cancer
Larsson [33]	SMC	USA	35,273	7.2	62.08	100	SSB	Pancreatic cancer
Larsson [33]	COSM	USA	42,524	7.2	59.7	0	SSB	Pancreatic cancer
Lee [34]	NHS	USA	68,738	20	52.98	100	SSB	Kidney cancer
Lee [34]	HPFS	USA	47,918	14	54.43	0	SSB	Kidney cancer
Nöthlings [35]	Multiethnic Cohort Study	USA	162,150	8	59.76	55.1	Fruit juice	Pancreatic cancer
Bao [36]	NIH-AARP Diet and Health Study	USA	487,922	7.2	62.17	0	SSB; ASB; Total soft drinks	Pancreatic cancer
Ren [37]	NIH-AARP Diet and Health Study	USA	481,563	NR	50–71	40.53	SSB	Oral; pharynx; larynx; esophagus; gastric cancer
Mueller [38]	SCHS:	Singapore	60,524	14	56.33	55.9	SSB; Fruit juice	Pancreatic cancer
Fung [39]	NHS + HPFS	USA	132,392	26	NR	100	SSB	Colorectal cancer
Zhang [40]	Pooled analysis	China	731,441	6–20	25–90	NR	SSB	Colon cancer
Ros [41]	EPIC	Europe	233,236	9.3	25–70	NR	Total soft drink	Urinary tract cancer
Allen [42]	Million Women Study	UK	779,369	5.2	59.4	100	SSB; Fruit juice	Kidney cancer
Friberg [43]	SMC	Sweden	61,226	18.4	53.83	NR	Total soft drink	Endometrial cancer
Drake [44]	MDC	Sweden	28,098	14.9	59.00	60.63	SSB; Fruit juice	Prostate cancer
Schernhammer, 2012 [18]	NHS	USA	77,218	22	53.70	100	SSB; ASB	Non-Hodgkin lymphoma; Myeloma; Leukemia
Schernhammer, 2012 [18]	HPFS	USA	47,810	22	50.60	0	SSB; ASB	Non-Hodgkin lymphoma; Myeloma; Leukemia
Inoue-Choi 2013 [45]	IWHS	USA	41,836	4	61.62	100	SSB; 100% Fruit juice	Endometrial Cancer
Stepien [46]	the European Prospective Investigation into Cancer and Nutrition cohort	Europe	477,206	11.4	51.21	70.2	Total soft drinks	Liver cancer
Odegaard [47]	SCHS	China	52,584	16.3	55.77	56.02	SSB; 100% Fruit juice	Overall cancer
McCullough [48]	CPS-II Nutrition Cohort	USA	100,442	10	69.20	56.84	SSB; ASB	Non-Hodgkin lymphoma
Wu [49]	HPFS	USA	41,530	24–26	53.31	0	Fruit juice	Basal cell carcinoma; squamous cell carcinoma
Wu [49]	NHS	USA	63,759		50.09	100	Fruit juice	Basal cell carcinoma; squamous cell carcinoma
Wu [50]	HPFS	USA	105,432	NR	NR	NR	Fruit juice	Melanoma
Hodge [51]	MCCS	Australia	35,393	11.6	54.69	60.38	SSB; ASB	Overall; Prostate; Ovary; Kidney; Colorectal; Breast; endometrial; Gastric cancer
Zamora-Ros [52]	EPIC	Europe	519,978	14	35–70	70	Fruit juice	Thyroid cancer
Miles [53]	PLCO	Canada	38,343	9	65.6	0	SSB; ASB; Fruit juice	Prostate Cancer
Makarem [54]	FOS	USA	3418	NR	54.34	52.98	SSB; Fruit juice	Breast; Prostate; Colorectal cancer
Bassett et al. [55]	MCCS	Australia/New Zealand/other, United Kingdom; Italy; Greece	35,109	19	54.7	60.69	SSB	Prostate; lymphoma; gastric; Melanoma; breast; bladder; Brain cancers; Leukemia
Luo et al. [56]	NHS	USA	87943	32	58.65	100	SSB	Liver cancer
Luo et al. [56]	HPFS	USA	49665			0	SSB	Liver cancer
Pacheco et al. [57]	CTS	USA	99798	20	51.58	100	SSB	Colorectal Cancer
Romanos-Nanclares [58]	SUN	Spain	10713	10	34.76	100	SSB	Breast cancer
Chazelas,2019 [13]	NutriNet-Santé prospective cohort	French	101257	5.1	42.2	78.7	SSB; ASB; Fruit juice	Overall; Breast; Colorectal; Prostate cancer
Debras [59]	NutriNet-Santé cohort	France	101,279	5.9	40.8	78.73	SSB	Breast cancer
	Hokkaido	Japan	3158	14.3	58.00	51.74	SSB	Overall, colorectal cancer
Heath [60]	EPIC	Europe	389,220	15	68.00	51.80	Total soft drink; SSB; ASB; Fruit juice	Kidney cancer
Arthur [61]	CSDLH	Canada	73,909	12	NR	53.60	SSB; Fruit juice	Breast, Endometrial, Ovarian, Colorectal cancer
Hur [62]	NHSII	USA	95,464	24	41.55	100	SSB; ASB, Fruit juice	Colorectal cancer
Chen [63]	HMAC	China	491,929	25	39.9	52.14	SSB	Pancreatic cancer
Romanos-Nanclares [64]	NHSII NHS	USA	90,085 82,713	26 36	36.5 46.2	100	SSB; ASB;	Breast cancer
Yuan [65]	HPFS NHS	USA	51,529 121,700	28 30	40–75 30–55	0 100	SSB	Overall, Colon, Rectum cancer
Ringel [66]	NR	USA	93,676	13.5	50–79	100	ASB	Urinary tract cancer

*EPIC* European Prospective Investigation into Cancer and Nutrition, *NHS* Nurses’ Health Study, *SCHS* Singapore Chinese Health Study, *CPS* Cancer Prevention Study, *IWHS* Iowa Women’s Health Study, *MDC* Malmo¨ Diet and Cancer, *SMC* Swedish Mammography Cohort, *COSM* Cohort of Swedish Men, *MCCS* Melbourne Collaborative Cohort Study, *CTS* California Teachers Study, *PLCO* Prostate, Lung, Colorectal, and Ovarian, *NCS* Nutrition Canada Survey, *FOS* The Framingham Offspring, *SUN* Seguimiento Universidad de Navarra, *HMSC* Half a Million Asian Cohort, *SSB* sugar sweetened beverages, *ASB* artificially sweetened beverages

### Risk of bias assessment

The details of the risk of bias assessment are presented in Supplementary Tables S5 and S6. Eighteen cohorts were considered to have a high risk of bias due to the retrospective assessment of exposure. Three cohorts were at high risk because of a lack of confidence that the outcome of interest was not present at the start of the study. Two cohorts had a high risk of bias in the outcome measures and confounder adjustment domains. Ten cohorts had a high risk of bias due to insufficient follow-up of cohorts. The other domains for all studies were at a low risk of bias. Overall, 9 cohorts were classified as having a high risk of bias.

### Consumption of SSBs and the risk of cancer

Thirty-two articles focused on the consumption of SSBs and the risk of cancer, of which 31 were included in the dose–response meta-analysis, and all 32 articles were included in the highest category versus the lowest category meta-analysis. With low to moderate certainty of evidence, an increment of 250 ml/day in SSB intake was associated with a 17% greater risk of breast cancer (RR = 1.17, 95% CI: 1.00 to 1.37; *I*^*2*^ = 66%; absolute risk difference 8 more per 1000 persons), a 10% greater risk of colorectal cancer (RR = 1.10, 95% CI: 1.04 to 1.15; *I*^*2*^ = 0%; absolute risk difference 2 more per 1000 persons), a 30% risk of biliary tract cancer (RR = 1.30, 95% CI: 1.10 to 1.54; absolute risk difference 1 more per 1000 persons), and a 10% risk of prostate cancer (RR = 1.10, 95% CI: 1.00 to 1.22; *I*^*2*^ = 0%; absolute risk difference 4 more per 1000 persons). An increment of 250 mL/day in SSB intake was not significantly associated with the risk of overall, endometrial, esophagus, gastric, hepatocellular, kidney, oral, ovarian, pancreatic, larynx or pharynx cancer, or leukemia, Multiple myeloma, non-Hodgkin lymphoma (Tables 2 and 3, and supplementary Table S7). For the lowest category versus the highest category comparison, the results from 32 studies were generally consistent with the finding from our dose–response meta-analysis (Appendix Figures S1 to S4).

**Table 2:** Results of beverages consumption (per 250 mL/day increase) and specific cancer (more than 2 studies) risk

Outcomes	Studies, n	Mean Follow-up, y	RR (95%CI)	Population risk per 1000 persons over 10.8 y^a^	Risk difference per 1000 person (95%CI)	GRADE certainty of evidence	Summary
**SSBs**							
Overall cancer	4	13.00	1.07 (0.95 to 1.22)	185	13 (-9 to 41)	Very low^b,d^	We are uncertain of the effects of Per 250 mL/day increase of SSBs consumption and all cancer risk
Breast cancer	7	8.15	1.17 (1.00 to 1.37)	46	8 (0 to 17)	Moderate^b,c^	Per 250 mL/day increase of SSBs consumption is likely to have small effect on breast cancer risk
Colorectal cancer	8	15.68	1.10 (1.04 to 1.15)	20	2 (1 to 3)	Moderate^b,c^	Per 250 mL/day increase of SSBs consumption is likely to have small effect on colorectal cancer risk
Endometrial cancer	2	7.80	1.01 (0.99 to 1.03)	10	0 (0 to 0)	Very low^b,d^	We are uncertain of the effects of Per 250 mL/day increase of SSBs consumption and endometrial cancer risk
Gastric cancer	2	11,6	1.00 (0.85 to 1.17)	14	0 (-2 to 2)	Very low^b,d^	We are uncertain of the effects of Per 250 mL/day increase of SSBs consumption and Gastric cancer risk
Kidney cancer	3	12.7	1.06 (0.98 to 1.15)	5	0 (0 to 1)	Low^b,c,d^	We are uncertain of the effects of Per 250 mL/day increase of SSBs consumption and kidney cancer risk
Leukemia	2	22	1.06 (0.73 to 1.54)	4	0 (-1 to 2)	Very low^b,d^	We are uncertain of the effects of Per 250 mL/day increase of SSBs consumption and Leukemia risk
Multiple myeloma	2	22	1.18 (0.90 to 1.55)	2	0 (0 to 1)	Very low^b,d^	We are uncertain of the effects of Per 250 mL/day increase of SSBs consumption and Multiple myeloma risk
Non-Hodgkin lymphoma	3	16	1.07 (0.92 to 1.23)	5	0 (0 to 1)	Very low^b,d^	We are uncertain of the effects of Per 250 mL/day increase of SSBs consumption and Non-Hodgkin lymphoma risk
Pancreatic cancer	7	11.33	1.08 (0.97 to 1.21)	5	0 (-1 to 2)	Low^b,d^	We are uncertain of the effects of Per 250 mL/day increase of SSBs consumption and pancreatic cancer risk
Prostate cancer	5	8.57	1.10 (1.00 to 1.22)	38	4 (0 to 8)	Low^b^	Per 250 mL/day increase of SSBs consumption is likely to have small effect on prostate cancer risk
**ASBs**							
Overall cancer	2	8.35	0.96 (0.86 to 1.08)	185	-7 (-26 to 15)	Very low^b,d^	We are uncertain of the effects of Per 250 mL/day increase of SSBs consumption and all cancer risk
Breast cancer	3	8.35	0.95 (0.80 to 1.12)	46	-1 (-2 to 0)	Very low^b,d^	We are uncertain of the effects of Per 250 mL/day increase of SSBs consumption and breast cancer risk
Colorectal cancer	2	11.6	0.93 (0.78 to 1.10)	20	-2 (-4 to 0)	Very low^b,d^	We are uncertain of the effects of Per 250 mL/day increase of SSBs consumption and Colorectal cancer risk
Multiple myeloma	2	22	1.14 (0.81 to 1.60)	2	0 (0 to 1)	Very low^b,d^	We are uncertain of the effects of Per 250 mL/day increase of SSBs consumption and multiple myeloma risk
Non-Hodgkin lymphoma	3	16	1.00 (0.90 to 1.11)	5	0 (-1 to 1)	Very low^b,d^	We are uncertain of the effects of Per 250 mL/day increase of SSBs consumption and non-Hodgkin lymphoma risk
pancreatic cancer	3	12.93	1.03 (0.96 to 1.10)	5	0 (0 to 1)	Low^b,c,d^	We are uncertain of the effects of Per 250 mL/day increase of SSBs consumption and pancreatic cancer risk
Prostate cancer	2	8.35	0.93 (0.69 to 1.26)	38	-3 (-12 to 10)	Very low^b,d^	We are uncertain of the effects of Per 250 mL/day increase of SSBs consumption and Prostate cancer risk
**100% Fruit juice**							
Overall cancer	2	10.7	1.31 (1.04 to 1.65)	185	57 (7 to 120)	Low^b^	Per 250 mL/day increase of SSBs consumption is likely to have small effect on all cancer risk
breast cancer	3	5.1	1.07 (0.96 to 1.18)	46	3 (-2 to 8)	Very low^b,d^	We are uncertain of the effects of Per 250 mL/day increase of SSBs consumption and breast cancer risk
Colorectal cancer	3	5.1	1.21 (1.00 to 1.47)	20	4 (0 to 9)	Very low^b,d^	We are uncertain of the effects of Per 250 mL/day increase of SSBs consumption and Colorectal cancer risk
endometrial cancer	2	5	1.05 (1.00 to 1.10)	10	1 (0 to 1)	Very low^b,d^	We are uncertain of the effects of Per 250 mL/day increase of SSBs consumption and endometrial cancer risk
pancreatic cancer	3	11.2	0.91 (0.61 to 1.35)	5	0 (-2 to 2)	Very low^b,c,d^	We are uncertain of the effects of Per 250 mL/day increase of SSBs consumption and pancreatic cancer risk
Prostate cancer	3	8.57	1.13 (0.93 to 1.39)	38	5 (-3 to 15)	Very low^b,d^	We are uncertain of the effects of Per 250 mL/day increase of SSBs consumption and Prostate cancer risk

*SSB* sugar-sweetened beverages, *ASB* artificially sweetened beverages, *RR* relative risk, *GRADE* Grading of Recommendations Assessment, Development and Evaluation, *CVD* cardiovascular disease, *SSBs* Sugar-sweetened beverages, *ASBs* Artificially sweetened beverages, *HR* Hazard ratios

^a^Population risk of cancer incidence comes from Lifetime cumulative risk from GLOBOCAN 2012

^b﻿^Certainty of evidence starts from low due to observational design

^c﻿^Upgraded one level as dose–response gradient is present

^d﻿^Downgraded one level for imprecision as confidence interval around absolute effect includes both small benefit and small harm

**Table 3:**
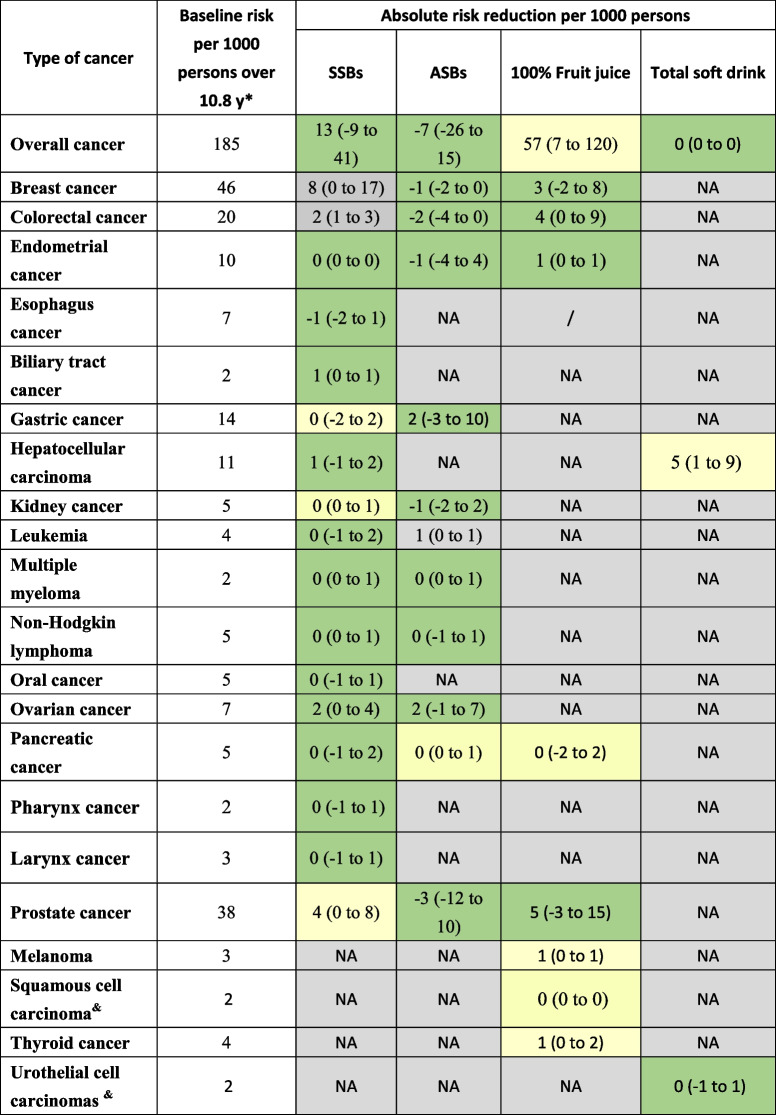
Summary of findings for beverages consumption (per 250 mL/day increase) and specific cancer risk

*SSB* sugar-sweetened beverages, *ASB* artificially sweetened beverages, *RR* relative risk, *GRADE* Grading of Recommendations Assessment, Development and Evaluation, *CVD* cardiovascular disease, *SSBs* Sugar-sweetened beverages, *ASBs* Artificially sweetened beverages, *HR* Hazard ratios

^*^Population risk of cancer incidence comes from Lifetime cumulative risk from GLOBOCAN 2012

^&^Population risk of cancer incidence comes from the cancer incidence of reference group

**Fig. 2:**
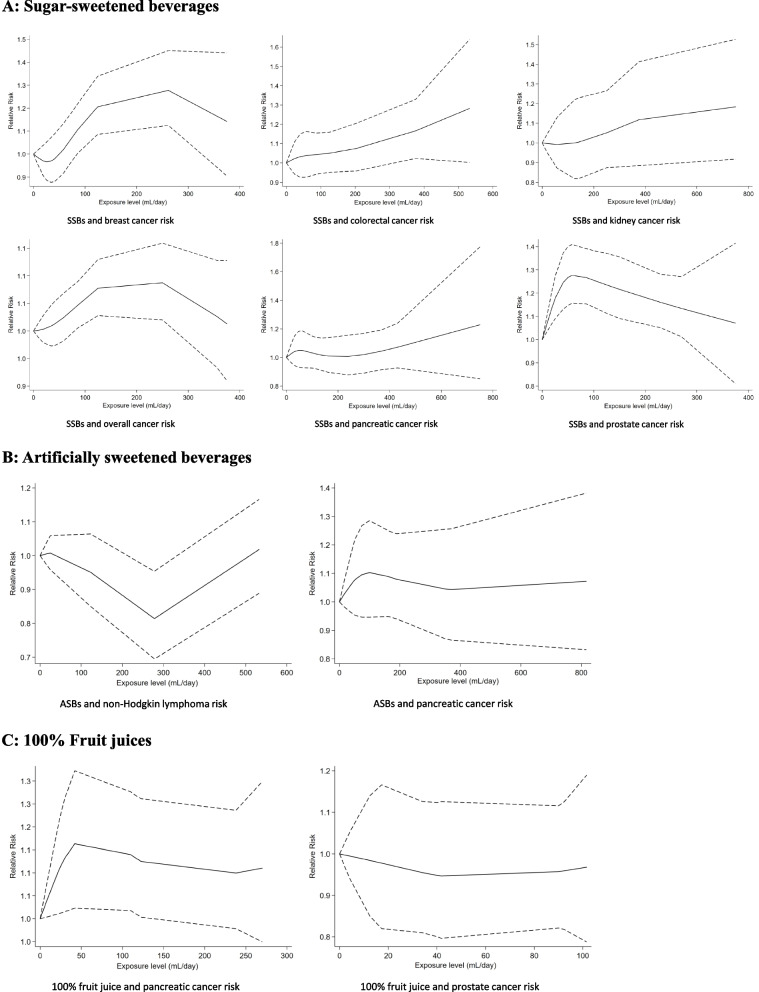
Dose–response association between SSBs, ASBs, and 100% fruit juices intake and cancer risk

There was a linear dose–response association between SSBs consumption and the risk of breast and kidney cancer (*P *_*non-linearity*_ = 0.6343, *P *_*non-linearity*_ = 0.185, respectively) (Fig. 2). We observed nonlinear dose–response associations between SSBs consumption and the risk of colorectal cancer (n = 8 studies, *P *_*non-linearity*_ = 0.0304), all cancer (n = 4 studies, *P *_*non-linearity*_ = 0.0770), prostate cancer (n = 5 studies, *P *_*non-linearity*_ = 0.0054), and pancreatic cancer (*n* = 7 studies, *P *_*non-linearity*_ = 0.0294).

Subgroup analyses (Supplementary table S8) showed that increased SSB consumption of 250 mL/day for participants from Asia and Europe had a stronger association with the risk of pancreatic cancer than those from the USA (*P *_*interaction*_ = *0.006*), and studies with shorter follow-up durations showed stronger association than those with longer follow-up durations (*P *_*interaction*_ = *0.03*). There were no significant subgroup effects in terms of age, sex, duration of follow-up, or risk of bias for other types of cancer.

We conducted a sensitivity analysis by excluding studies [67] that reported the risk of cancer for SSBs combined with 100% fruit juice rather than for SSBs alone. This result was consistent with that of the primary analysis (RR = 1.17, 95% CI: 1.00 to 1.38).

### Consumption of ASBs and the risk of cancer

Eleven articles reported the consumption of ASBs and the risk of cancer, and seven were included in the dose–response meta-analysis, and all of 11 articles were included in the highest category versus lowest category meta-analysis. Low certainty of evidence showed that increment ASB of 250 mL/day was associated with a 16% greater risk of leukemia (RR = 1.16, 95% CI: 1.00 to 1.35; *I*^*2*^ = 0%; absolute risk difference 1 more per 1000 persons). All 11 studies were included in the highest category versus lowest comparison. We did not find any statistically significant differences between ASB consumption per 250 mL/day and the risk of other types of cancer (Tables 2 and 3, supplementary Table S7). The results of the highest versus lowest meta-analyses of ASBs intake and the cancer risk were similar to those of the dose–response meta-analysis (Appendix Figures S1 to S4).

There was a linear dose–response association between ASBs intake and the risk of pancreatic cancer (*n* = 3 studies, *P *_*non-linearity*_ = 0.3225) (Fig. 2).

We did not find any statistically significant subgroup effects for any factors.

### Consumption of 100% fruit juices and the risk of cancer

In total, 16 articles reported on the consumption of 100% fruit juice and the risk of cancer. All these articles were included in the dose–response meta-analysis and highest category versus lowest category meta-analysis. An intake increase of 250 mL of 100% fruit juice per day was associated with 31% increase in the risk of overall cancer (RR = 1.31, 95% CI: 1.04 to 1.65; *I*^*2*^ = 70.6%; absolute risk difference 57 per 1000 persons) and 22% risk of melanoma (RR = 1.22, 95% CI: 1.14 to 1.31; absolute risk difference 1 more per 1000 persons), 2% risk of squamous cell carcinoma (RR = 1.02, 95% CI: 1.00 to 1.04; absolute risk difference 0 more per 1000 persons), and 28% risk of thyroid cancer RR = 1.28, 95% CI: 1.08 to 1.53; absolute risk difference 1 more per 1000 persons) (Tables 2 and 3, supplementary Table S7). We found that the results of highest versus lowest comparisons were consistent with the dose–response meta-analysis.

There was a linear dose–response association between 100% fruit juice intake and the risk of pancreatic cancer (*n* = 3 studies, *P *_*non-linearity*_ = 0.9597) (Fig. 2). We observed a nonlinear dose–response association between 100% fruit juice intake and the risk of prostate cancer (*n* = 3 studies, *P *_*non-linearity*_ = 0.0618) (Fig. 2).

We did not find any a statistically significant subgroup effect for any factors.

### Consumption of total soft drink and the risk of cancer

Five articles focused on the relationship between total soft drink consumption and cancer risk. All five articles were included in the dose–response meta-analysis and the highest category versus lowest category meta-analysis. An intake of total soft drink per 250 mL/day was only associated with the risk of hepatocellular carcinoma (RR = 1.42, 95% CI: 1.08 to 1.87; absolute risk difference 5 more per 1000 persons) (Table 2 and supplementary Table S7). The results of the highest versus lowest comparisons were similar to those of the dose–response meta-analysis (Appendix Figures S1 to S4).

We did not find any a statistically significant subgroup effect for any factors.

## Discussion

### Principle findings

This systematic review and meta-analysis included 42 articles with 4,518,547 participants. Overall, the impacts of soft drinks on the risk of different types of cancer was small (less than 10 increased per 1000 persons). Dose–response meta-analyses showed that with low to moderate certainty evidence, an SSBs intake increase of 250 mL per day might result in 8 more per 1000 persons for breast cancer, 2 more per 1000 persons for colorectal cancer, and 1 more per 1000 persons for biliary tract cancer; ASBs intake increase of 250 mL per day might result in 1 more per 1000 persons in leukemia; and 100% fruit juices intake increase of 250 mL per day might result in 52 more per 1000 persons in overall cancer, 1 more per 1000 persons in melanoma and thyroid cancer each. Subgroup analyses showed that increased SSB consumption of a 250 mL/day in participants from Asia and Europe had a stronger association with the risk of pancreatic cancer than those from the USA, and studies with shorter follow-up duration showed stronger association than those with longer follow-up duration. We found linear dose–response associations between the consumption of SSBs and the risk of breast and kidney cancer, and between the consumption of ASBs and 100% fruit juices and the risk of pancreatic cancer. For SSBs consumption and the risk of colorectal, overall, prostate, and pancreatic cancer, as well as 100% fruit juice intake and the risk of prostate cancer, we observed non-linear dose–response associations.

### Comparison with other studies

Our findings confirm or refute several previously published systematic reviews. The results of our study on colorectal cancer differ from those of previous systematic reviews and meta-analysis. Schwingshackl and colleagues [68] found that there was no association between colorectal cancer and the SSBs consumption (2 studies with 863,833 participants, RR = 1.09, 95% CI = 0.97 to 1.12). In contrast, our study observed a positive association between SSBs consumption and risk of colorectal cancer. Our results regarding the risk of prostate, bladder, pancreatic, esophageal, colon, or kidney cancer were consistent with those of previous meta-analyses. Gallus et al. conducted a systematic review [69], that included five cohort studies with 929,709 participants and concluded that increased consumption of SSBs was not associated with the risk of pancreatic cancer (RR: 1.05, 95%CI: 0.94 to 1.17). Boyle and colleagues [70] found no association between high SSB consumption and increased incidence of prostate, esophageal, pancreatic, kidney, colon, or bladder cancer. However, above studies were limited by pooling the results relied on extreme exposure categories, and did not consider the certainty of evidence and absolute effects. Llaha and colleagues [19] conducted a systematic review to investigate the associations between SSBs, ASBs, and fruit juices and cancer incidence. They found that SSBs consumption was associated with breast cancer, which is consistent with our findings. They also found that SSBs and fruit juices intake were associated with the risk of prostate cancer, which were not found in our review. However, their systematic review was limited by the fact that their results was based only on extreme exposure categories and failed to investigate the dose–response association, and they also did not provide the results of certainty of evidence. In terms of biliary tract cancer, leukemia, and melanoma, our review firstly investigated the association between SSBs, ASBs, and 100% fruit juice and above factors, and we found positive association. Our systematic review could provide up-to-date evidence for soft drink and 100% fruit juices, including the dose–response association, the absolute effect, and certainty of evidence for each outcome of specific cancer risk. A report of systematic review and meta-analysis published by WHO [71] suggested that ASBs was associated with 31% increased risks of bladder cancer but not risk of other type of cancer, which was inconsistent with findings from our systematic review. The main reason that could explain above differences may that the different eligibility criteria, type and number of included studies, and differences of the methods used among reviews.

### Strengths and limitations

Our systematic review has several strengths. Our review is the first to address the dose–response associations between SSBs, ASBs, 100% fruit juices, or total soft drink consumption and specific cancer risk. We focused not only on meta-analysis of highest versus lowest comparison but also dose–response meta-analysis, which provides the most convincing evidence for the associations between SSBs, ASBs, 100% fruit juices, or total soft drink and health outcomes. We conducted of this systematic review and meta-analysis according PRISMA checklist to ensure higher reporting quality [72] and we evaluated the certainty of evidence using the GRADE approach for SSBs, ASBs, 100% fruit juices, or total soft drinks intake and cancer risk, thus highlighting the remaining uncertainty regarding causal relationships between sweetened beverage consumption and cancer. Furthermore, we presented the results with an absolute effect, which is an easy-to-understand approach [73]. In addition, our analysis was based on a large number of participants and therefore provided sufficiently reliable estimates for some types of cancer.

Limitations also existed in this systematic review. First, our review included prospective cohort studies, which are observational studies that are prone to confounding. Second, our study identified 42 studies that focused on 22 types of cancer, of these, 11 types of cancer only included one studies, which may make the results of our analyses that focused on above 11 types of cancer insufficiently informative and relative lower power of the analyses. More cohort studies are needed to further investigate the associations between soft drinks and the risk of cancers, and more randomised controlled trials are needed to confirm the adverse health effects of soft drinks. Third, the dietary information was assessed mainly based on questionnaire in included cohorts, which might result in recall bias. Fourth, servings are usually different depending on the country, in our study, we used a median intake of 250 mL/serving, which could be underestimating the intake of SSBs, ASBs, and 100% fruit juices. Fifth, dietary exposure is prone to be correlated with other potentially confounding factors, however, among eligible studies, there was a lack of adequate adjustment for potential confounders (such as adjustment of other beverages, BMI, alcohol consumption, aspirin use, or socioeconomic status, et al.), which is a major source of potential bias [74].

### Implications

SSBs usually contain 1 to 12 percent sugar, which may result in adverse health effects [9]. Consumption of SSBs and the risk of cancer may partly attribute to overweight and obesity, which is one of major risk factor for ovarian, stomach, oral, pancreatic, breast, gallbladder, larynx, pharynx, liver, prostate, kidney, endometrial, and colorectal cancers [75]. Additionally, fructose is main natural carbohydrate sweetener of SSBs in USA, overconsumption of fructose could affect liver fat formation through intestinal flora, which might promote tumorigenesis through alterations in adipokine secretion and cell signaling pathways [76–79]. Considering the adverse health effects of SSBs, artificial sweeteners are commonly preferred to be added in beverages, and labelled as ‘no added sugar’. However, artificial sweeteners can increase the desire for sweet taste [80], leading to excessive consumption of calories, weight gain, and an increased risk of disturbed glucose homeostasis [80]. The higher risk of leukemia observed in this review in those who consumed ASBs supports the hypothesis of the adverse effects of ASBs. Fruit juice is high in naturally occurring sugar and has less dietary fiber than that in whole fruit and may provide extra dietary calories [11], and thus, it is widely emphasised from the American Academy of Pediatrics (AAP), US Department of Health and Human Services and US Department of Agriculture (DGA), and the Robert Wood Johnson Foundation Healthy Eating Research program [67, 81] that water and whole fruit are preferred to fruit juice. Therefore, the results of our study support the concept that the risk of cancer could be associated with a higher consumption of SSBs, ASBs, or fruit juice, and the existing nutritional recommendations to limit soft drink consumption [75]. The implementation action of limit soft drink consumption policy could potentially contribute to the reduction in cancer incidence [75, 82]. More targeted intervention, action-oriented research, and public education of the above factors may be appropriate.

## Conclusion

We demonstrated that an increment of 250 mL/day in SSB intake was positively associated with risk of breast, colorectal and biliary tract cancers, but the evidence was graded as being of low certainty. The consumption of 100% fruit juices was also positively associated with overall cancer and thyroid cancer risk, and the risk of melanoma. The association of ASBs consumption with specific cancer risk was uncertain except for leukemia. More targeted intervention, action-oriented research, and public education of the above factors may be appropriate.

## Supplementary Information

**Additional file 1. **Supplementary material is available at QJMED online.

## Data Availability

All data and materials have open access to the public upon reasonable requests.

## Abbreviations

SSB: Sugar-sweetened beverage
ASB: Artificially sweetened beverage
GRADE: Grading of Recommendations Assessment, Development and Evaluation
RR: Relative risks
HR: Hazard ratio
CI: Confidence Interval
