# Supplementary material for: Association of soft drinks and 100% fruit juice consumption with risk of cancer: a systematic review and dose–response meta-analysis of prospective cohort studies
Source: Int J Behav Nutr Phys Act. 2023 May 15;20:58. doi: 10.1186/s12966-023-01459-5 (PMC10184323; doi:10.1186/s12966-023-01459-5)
Supplement: Supplementary file 1 — Additional file 1. Supplementary material is available at QJMED online. [file 12966_2023_1459_MOESM1_ESM.docx]

**Association of soft drink and 100% fruit juice consumption with risk of cancer: a systematic review and dose-response meta-analysis of prospective cohort studies**

[Table S1 Search strategy 2](#_Toc129791974)

[Table S2 Definitions of exposures 6](#_Toc129791975)

[Table S3 Study eligibility form 7](#_Toc129791976)

[Table S4 Baseline characteristics of included studies 9](#_Toc129791977)

[Table S5 Detailed guidance for assessment of risk of bias 24](#_Toc129791978)

[Table S6 Results of risk of bias assessment 28](#_Toc129791979)

[Table S7 Summary of findings for beverages consumption (per 250mL/day increase) and specific cancer risk 31](#_Toc129791980)

[Table S8 Summary results of dose-response meta-analyses, subgroup analyses (increase 250ml per day) 44](#_Toc129791981)

[Figure S1 Results of Sugar-sweetened beverages consumption and the risk of cancer incidence in highest versus lowest comparison 48](#_Toc129791982)

[Figure S2 Results of Artificially sweetened beverages consumption and the risk of cancer incidence in highest versus lowest comparison 49](#_Toc129791983)

[Figure S3 Results of 100% Fruit Juices consumption and the risk of cancer incidence in highest versus lowest comparison 50](#_Toc129791984)

[Figure S4 Results of Total soft drinks consumption and the risk of cancer incidence in never versus ever comparison 51](#_Toc129791985)

[Appendix 1 the lists of studies excluded in full-text screen 52](#_Toc129791986)

[Appendix 2 the lists of included studies 61](#_Toc129791987)

[Appendix 3 the PRISMA checklist 64](#_Toc129791988)

# Table S1 Search strategy

Database: *PubMed*

Search Strategy:

| **#** | **Searches** |
| --- | --- |
| #1 | "Carbonated Beverages"(Mesh) |
| #2 | "sugar drink*"(Title/Abstract) OR "Carbonated Water"(Title/Abstract) OR "Energy Drinks"(Title/Abstract) |
| #3 | SSBs (Title/Abstract) OR SSB (Title/Abstract) OR "sugar-sweetened beverage" (Title/Abstract) OR "sugar-sweetened beverages" (Title/Abstract) OR "sugar sweetened beverage" (Title/Abstract) OR "sugar sweetened beverages" (Title/Abstract) |
| #4 | lemonade(Title/Abstract) OR lemonades(Title/Abstract) |
| #5 | "soft drink"(Title/Abstract) OR "soft drinks"(Title/Abstract) OR soft-drinks(Title/Abstract) OR soft-drink(Title/Abstract) |
| #6 | pop(Title/Abstract) OR soda(Title/Abstract) OR "soda pop"(Title/Abstract) |
| #7 | ASB (Title/Abstract) OR ASBs (Title/Abstract) OR "artificially sweetened beverages" (Title/Abstract) OR "artificially sweetened beverage" (Title/Abstract) OR "artificially-sweetened beverages" (Title/Abstract) OR "artificially-sweetened beverage" (Title/Abstract) |
| #8 | ((“fruit juice*”(Title)) OR beverage*(Title)) OR juice*(Title) |
| #9 | #1 OR #2 OR #3 OR #4 OR #5 OR #6 OR #7 OR #8 |
| #10 | "neoplasms"(Mesh) |
| #11 | "cancer*" (Title/Abstract) or "carcinoma*"(Title/Abstract) |
| #12 | "leukemia"(MeSH Terms) |
| #13 | leukemia(Title/Abstract) |
| #14 | #10 OR #11OR #12 OR #13 |
| #15 | "Cohort Studies"(Mesh) |
| #16 | "Longitudinal Studies"(Mesh) |
| #17 | longitudinal (Title/Abstract) OR cohort (Title/Abstract) OR cohorts (Title/Abstract) OR follow-up (Title/Abstract) OR case-cohort (Title/Abstract) |
| #18 | predict*(Title) OR associat*(Title/Abstract)) OR risk(Title/Abstract) |
| #19 | "Risk"(Mesh) |
| #20 | "Survival Analysis"(Mesh) |
| #21 | regression*(Title/Abstract)) OR survival analysis(Title/Abstract) |
| #22 | "Regression Analysis"(Mesh) |
| #23 | #15 OR #16 OR #17 OR #18 OR #19 OR #20 OR #21 OR #22 |
| #24 | #9 AND #14 AND #23 |

Database: *Cochrane*

Search Strategy:

| **#** | **Searches** |
| --- | --- |
| #1 | MeSH descriptor: (Carbonated Beverage) explode all trees |
| #2 | (“drinking water”):ti,ab,kw OR (“Carbonated Water”) :ti,ab,kw OR (“Energy Drink*”):ti,ab,kw |
| #3 | (SSB):ti,ab,kw OR (SSBs):ti,ab,kw OR (sugar-sweetened beverage):ti,ab,kw OR (sugar-sweetened beverages):ti,ab,kw OR (sugar-sweetened):ti,ab,kw |
| #4 | (lemonade):ti,ab,kw OR (lemonades):ti,ab,kw |
| #5 | (soda):ti,ab,kw OR (pop):ti,ab,kw OR OR (soda pop):ti,ab,kw |
| #6 | (“soft drink”): ti,ab,kw OR (“soft drinks”): ti,ab,kw OR (soft-drink): ti,ab,kw OR (soft-drinks): ti,ab,kw |
| #7 | (ASB): ti,ab,kw OR (ASBs): ti,ab,kw OR (artificially sweetened beverages): ti,ab,kw OR (artificially sweetened beverage): ti,ab,kw |
| #8 | (“Fruit juices”): ti,ab,kw OR (“Fruit juice”): ti,ab,kw OR (juice): ti,ab,kw OR (juices): ti,ab,kw |
| #9 | (beverage): ti,ab,kw OR (beverages): ti,ab,kw |
| #10 | #1 OR #2 OR #3 OR #4 OR #5 OR #6 OR #7 OR #8 OR #9 |
| #11 | MeSH descriptor: (neoplasms) explode all trees |
| #12 | (cancer*): ti,ab,kw |
| #13 | (carcinoma*): ti,ab,kw |
| #14 | (leukemia): ti,ab,kw |
| #15 | MeSH descriptor: (leukemia) explode all trees |
| #16 | #11 OR #12 OR #13 OR #14 OR #15 |
| #17 | MeSH descriptor: (Cohort Studies) explode all trees |
| #18 | MeSH descriptor: (Longitudinal Studies) explode all trees |
| #19 | (predict*):ti,ab,kw OR (predict):ti,ab,kw OR (associat*):ti,ab,kw OR (association):ti,ab,kw OR (risk):ti,ab,kw |
| #20 | MeSH descriptor: (Risk) explode all trees |
| #21 | MeSH descriptor: (Survival Analysis) explode all trees |
| #22 | MeSH descriptor: (Regression Analysis) explode all trees |
| #23 | (regression):ti,ab,kw OR (survival analysis):ti,ab,kw OR (regression):ti,ab,kw |
| #24 | (longitudinal):ti,ab,kw OR (cohort):ti,ab,kw OR (cohorts):ti,ab,kw OR (follow-up):ti,ab,kw OR (“follow up”):ti,ab,kw |
| #25 | #17 OR #18 OR #19 OR #20 OR #21 OR #22 OR #23 OR #24 |
| #26 | #10 AND #16 AND #25 |

Database: *Embase*

Search Strategy:

| **#** | **Searches** |
| --- | --- |
| #1 | 'carbonated beverages'/exp OR 'carbonated beverages' |
| #2 | 'drinking water':ab,ti OR 'carbonated water':ab,ti OR 'energy drink*':ab,ti |
| #3 | ssbs:ab,ti OR ssb:ab,ti OR 'sugar-sweetened beverage':ab,ti OR 'sugar-sweetened beverages':ab,ti OR 'sugar sweetened beverages':ab,ti OR 'sugar sweetened beverage':ab,ti |
| #4 | 'soft drinks':ab,ti OR 'soft drink*':ab,ti |
| #5 | pop:ab,ti OR soda:ab,ti OR 'soda pop':ab,ti |
| #6 | asb:ab,ti OR 'artificially sweetened beverages':ab,ti OR 'artificially sweetened beverage':ab,ti |
| #7 | lemonade:ab,ti OR lemonades:ab,ti |
| #8 | 'fruit juices':ab,ti OR 'fruit juice':ab,ti OR juice:ab,ti OR juices:ab,ti OR beverages:ab,ti OR beverage:ab,ti |
| #9 | #1 OR #2 OR #3 OR #4 OR #5 OR #6 OR #7 OR #8 |
| #10 | 'neoplasms'/exp OR neoplasms |
| #11 | 'leukemia'/exp OR leukemia |
| #12 | cancer*:ab,ti |
| #13 | carcinoma*:ab,ti |
| #14 | leukemia:ab,ti |
| #15 | #10 OR #11 OR #12 OR #13 OR #14 |
| #16 | 'cohort studies'/exp OR 'cohort studies' |
| #17 | 'risk'/exp OR risk |
| #18 | 'survival analysis'/exp OR 'survival analysis' |
| #19 | 'regression analysis'/exp OR 'regression analysis' |
| #20 | predict*:ab,ti OR associat*:ab,ti OR association:ab,ti OR risk:ab,ti |
| #21 | 'longitudinal studies'/exp OR 'longitudinal studies' |
| #22 | longitudinal:ab,ti OR cohort:ab,ti OR cohorts:ab,ti OR 'follow up':ab,ti OR 'case cohort':ab,ti OR 'nested case-control':ab,ti |
| #23 | #16 OR #17 OR #18 OR #19 OR #20 OR #21 OR #22 |
| #24 | #9 AND #15 AND #23 |

Database: *Web of Science*

Science Citation Index Expanded (SCI-EXPANDED) – from 1995 to now

Social Sciences Citation Index (SSCI) – from 1990 to now

Search Strategy:

| **#** | **Searches** |
| --- | --- |
| #1 | TS=("Drinking Water" OR "Carbonated Water" OR "Energy Drinks") |
| #2 | TS=(SSBs OR SSB OR "sugar-sweetened beverage" OR "sugar-sweetened beverages" OR "sugar sweetened beverage" OR "sugar sweetened beverages") |
| #3 | TS="Carbonated Beverage" |
| #4 | TS=(lemonade OR lemonades) |
| #5 | TS=("soft drink" OR "soft drinks" OR soft-drinks OR soft-drink) |
| #6 | TS=(pop OR soda OR "soda pop") |
| #7 | TS=(ABS OR "artificially sweetened beverages" OR "artificially sweetened beverage" OR "artificially-sweetened beverages" OR "artificially-sweetened beverage") |
| #8 | TS=("fruit juice" OR "fruit juices" OR juice OR fruit juices OR beverage OR beverages) |
| #9 | #1 OR #2 OR #3 OR #4 OR #5 OR #6 OR #7 OR #8 |
| #10 | TS=(neoplasms ) |
| #11 | TS=( leukemia) |
| #12 | TS=( cancer) |
| #13 | TS=(carcinoma) |
| #14 | #10 OR #11 #12 OR #13 |
| #15 | TS=("Cohort Studies") |
| #16 | TS=("Longitudinal Studies") |
| #17 | TS=(longitudinal OR cohort OR cohorts OR follow-up OR case-cohort) |
| #18 | TI=(predict* OR associat* OR risk) |
| #19 | TS=(regression OR "survival analysis" OR "Regression Analysis") |
| #20 | #15 OR #16 OR #17 OR #18 OR #19 |
| #21 | #9 AND #14 AND #20 |

# Table S2 Definitions of exposures

| **Type of beverages** | **Definition** |
| --- | --- |
| Sugar-sweetened beverages | Refer to any beverage with added sugar or other sweeteners (high fructose corn syrup, sucrose, fruit juice concentrates, and more). This includes soda, pop, caffeinated colas, caffeine-free colas, other (ie, noncola) carbonated sugar-sweetened beverages, and noncarbonated sugar-sweetened beverages, tonic, fruit punch, lemonade (and other “ades”), sweetened powdered drinks, as well as sports and energy drinks. |
| Artificially sweetened beverages | Defined as caffeinated, caffeine free, and noncarbonated low-calorie or diet beverages (ie. diet coke or diet fruit drinks) |
| 100 % Fruit juice | Defined as 100% fruit juice, or fruit juice assessed separately from soft drinks. |

# Table S3 Study eligibility form

| **Type of article:** | | |
| --- | --- | --- |
| - Cohort studies | **YES** | **NO** |

| **Type of participants:** | | |
| --- | --- | --- |
| - General health population that less than 20% of samples in cohorts had major chronic illness at the baseline | **YES** | **NO** |

| **Type of Exposure:** | | |
| --- | --- | --- |
| - Sugar-sweetened beverages (SSB) - Artificially sweetened beverages (ASB) - Fruit juice | **YES** | **NO** |

| **Type of Outcome:** | | |
| --- | --- | --- |
| - Risk of all types of cancer or mortality from cancer | **YES** | **NO** |

| **Study inclusion:** |  |
| --- | --- |
| - All the answers are YES | **INCLUDE** |
| - Any answer is NO | **EXCLUDE** |
| - If you are unsure of the answer, include for full text screening | **INCLUDE** |

**Instruction**

1. We will include Cohort studies that assessed the association between soft drink consumption and the risk of any types of cancer or mortality from cancer
2. We will consider study using multivariable analysis (Cox proportional hazards models, logistic regression models)
3. We will include conference abstracts when they reported the details on definition of soft-drink, adjusted factors, and results.
4. Considered as “YES” when some studies only mentioned the association between sugar source and the risk of cancer in title and abstract for full-text screen.
5. Considered as “YES” when studies only mentioned the association of sugar beverage or beverage consumption with the risk of cancer in title and abstract for full-text screen.
6. Soft-drink could be classified as sugar-sweetened beverages (SSB) and artificially sweetened beverages (ASB)
7. Sugar-sweetened beverages refer to any beverage with added sugar or other sweeteners (high fructose corn syrup, sucrose, fruit juice concentrates, and more). This includes soda, pop, caffeinated colas, caffeine-free colas, other (ie, noncola) carbonated sugar-sweetened beverages, and noncarbonated sugar-sweetened beverages, tonic, fruit punch, lemonade (and other “ades”), sweetened powdered drinks, as well as sports and energy drinks. were defined as caffeinated colas, caffeine-free colas, othern(ie, noncola) carbonated sugar-sweetened beverages, and noncarbonated sugar-sweetened beverages (fruit punches, soda, lemonades, or other fruit drinks, and so on).
8. Artificially sweetened beverages were defined as caffeinated, caffeine free, and noncarbonated low-calorie or diet beverages (ie. diet coke or diet fruit drinks)
9. 100% fruit juices were defined as 100% fruit juice, or fruit juice assessed separately from soft drinks.

# Table S4 Baseline characteristics of included studies

| **Author, year** | **Population Description** |  | **Beverage consumption** | | **Outcome** | | **Adjustment** |
| --- | --- | --- | --- | --- | --- | --- | --- |
|  |  | **Types of exposure** | | **Method of ascertainment** | **Types of outcomes** | **Method of ascertainment** |  |
| Odegaard, 2014 | General population | SSB; 100% fruit juice | | SFFQ | Overall cancer | ICD-9 codes 140.0–195.8 and 199–208.9 | Age, sex, dialect, education, yearof interview, smoking, moderate and vigorous activity, sleep, BMI,hypertension (except for cancer), nonbeverage vegetable-fruit-soy–rich dietary pattern score, andenergy intake |
| McCullough,  2014 | General population | SSB; ASB | | FFQ | Lymphoid Neoplasms | ICD-O-2 and ICD-O-3 | Age at baseline, gender, history of diabetes, BMI, smoking status, energy intake, and ASB or SSB |
| Choi,2013 | Postmenopausal  Women | SSB; 100% fruit juice | | FFQ | Endometrial Cancer | ICD)-10 codes: c.54–c.55 | Age, smoking, physical activity, alcohol use, estrogen use, age at menarche, age at menopause, number of live births, history of diabetes, and coffee intake |
| Drake, 2012 | General population | SSB; fruit juice | | Modified diet history method | Prostate cancer | ﻿Swedish Cancer Register | Age, year of study entry, season of data collection, energy intake, height, waist, physical activity, smoking, educational level, birth in Sweden, alcohol, calcium, and selenium. |
| Schernhammer, 2012 | General population | SSB; ASB | | FFQ | Non-Hodgkin lymphoma; Multiple myeloma; Leukemia | Identified incident cancers from state tumor registries;deaths that were ascertained from family members, the postal service, and the National  Death Index | Age; questionnaire cycle; sugar-sweetened soda consumption; fruit and vegetable consumption; multivitamin use;intakes of alcohol, saturated fat, animal protein, and total energy; race; BMI; height; discretionary physical activity; smoking history; and menopausal status. |
| Ren, 2010 | General population | SSB | | FFQ | Oral cavity; pharynx; larynx; esophagus; gastric cancer | ﻿ICD-O, third edition | Age, sex, tobacco smoking, alcohol drinking, BMI, education, ethnicity, usual physical activity throughout the day, vigorous physical activity, and the daily intake of fruit, vegetables, red meat, white meat, and calories. |
| Mueller, 2010 | General population | SSB; fruit juice | | FFQ | Pancreatic cancer | Singapore Cancer Registry and the Singapore Registry of Births and Deaths. | Age, sex,ethnicity, and year of interview, education, smoking index, moderate physical activity, alcohol, added sugar and candy, and total calories and BMI |
| Nöthlings, 2007 | General population | fruit juice | | FFQ | pancreatic cancer | ﻿ICD-02 codes  C25.0-C25.3 and C25.7-C25.9 | ﻿Age, pack-years of smoking, family history of pancreatic cancer, energy intake, intakes of red meat and  processed meat, and BMI. |
| Larsson, 2006 | General population | SSB | | FFQ | pancreatic cancer | ﻿(ICD-9) code 157 | Age, sex, education, smoking status and pack-years  , BMI, and intakes of total energy and alcohol |
| Lee, 2006 | General population | SSB | | SFFQ | Renal cell cancer | National Death Index | BMI, history of hypertension, parity, history of diabetes, smoking status, and total energy intake |
| Schernhammer, 2005 | General population | SSB; ASB | | FFQ | pancreatic cancer | National Death Index | Age, gender, follow-up cycle, history of diabetes, smoking status, quintiles of caloric intake, quintiles of nonvigorous physical activity, and other soft drink consumption, BMI. |
| Bassett, 2019 | General population | SSB | | FFQ | Prostate; Diffuse large B-cell lymphoma; Noncardia gastric;Melanoma; Premenopausal breast; bladder; Brain; Unknown Primary; Lymphoid leukaemia; Other; All nonobesity-related cancers | ﻿the Victorian Cancer Registry and the Australian  Institute of Health and Welfare, including the National Death Index and  the Australian Cancer Database. | ﻿Alcohol intake, country of birth, Mediterranean diet score, physical activity, socioeconomic position, sex and smoking status, the same confounders as the sugar-sweetened soft drink model and frequency of sugar-sweetened soft drink consumption. |
| Luo, 2019 | General population | SSB | | SFFQ | Hepatocellular carcinoma | ﻿ICD, Eighth  Revision, code 571 | ﻿Age, study period, gender, race, physical activity, smoking status, aspirin use, alcohol intake, and total calorie intake |
| Pacheco, 2019 | General population | SSB | | FFQ | Colorectal Cancer | ﻿linkage with the California Cancer Registry | ﻿Age, race/ethnicity, SES, total years smoked, alcohol intake, family history of colon cancer, history of polyps, diabetes, MVPA, aspirin use, multivitamin use, menopausal status and menopausal hormone therapy  use, and oral  contraceptive use, BMI, total energy intake, and a set of dietary intake covariates: red meat, processed meat, and non-starchy vegetable. |
| Hodge, 2017 | General population | SSB; ASB | | FFQ | total cancer; Prostate cancer; Ovary; Kidney cancer; Colorectal cancer; Breast cancer; endometrial cancer; Gastric cancer. | Cancer Registry or the Australian Cancer Database | ﻿SEIFA, country of birth, alcohol intake, smoking status, physical activity, Mediterranean diet core,sugar-sweetened soft drink consumption and waist circumference. |
| Miles 2018 | General population | SSB, Fruit juice | | FFQ | Prostate cancer | National Cancer Institute | Age, race, study center, BMI, education, smoking, family history of prostate cancer, history of diabetes, PSA screening, and energy intake |
| Zamora-Ros, 2017 | General population | Fruit juice | | FFQ | thyroid cancer | ﻿Cancer incident cases were identified through regional/national  cancer registries, except for German, Greek, and French centres. | ﻿Age, sex, BMI, smoking, education, physical activity, total energy and alcohol  intake. In women, also adjusted for menopausal status and type, oral contraceptive use, and infertility problems. |
| Larsson, 2016 | General population | SSB | | FFQ | Biliary tract cancer; Gallbladder cancer | ﻿Swedish Cancer Register | ﻿Age, sex, education, smoking, body mass index, dietary protein intake, and total energy intake. |
| Ellison,2000 | General population | SSB | | Dietary interview | Prostate cancer | ICO-8 | Age, coffee, cola, total alcohol, beer, wine, spirits, smoking status, pack-years smoking, BMI, highest educational level attained, respondent status, and intake of fat, fibre and calories as measured by a 24h food frequency questionnaire. |
| Debras, 2020 | General population | SSB | | Self-administrated questionnaires | Breast cancer | ICD-10 | Age, BMI, height, physical activity, smoking status, educational level, number of 24-h dietary records, family history of cancer, energy intake without alcohol, alcohol intake, sodium, saturated fatty acids, fiber intake and “Healthy” and “Western” dietary patterns age at menarche, age at first child, number of biological children, baseline menopausal status, oral contraceptive use at baseline and during follow-up, and hormonal treatment for menopause at baseline and during follow-up |
| Makarem ,2018 | General population | SSB; Fruit juice | | FFQ | Adiposity-related cancers; Breast cancer; Prostate cancer; Colorectal cancer | ﻿ICD-O | ﻿Age, sex, smoking, alcohol, energy, ﻿menopausal status, hormone therapy (HT), age at menopause, and  number of live births, total fiber and for red and processed  meat intake. |
| Navarrete-Muñoz,2016 | General population | SSB; ASB; Fruit juice; Total soft drinks | | Country-specific validated dietary questionnaires | Pancreatic cancer | ﻿International Classification of Diseases for Oncology, Third Edition codes C25.0–  C25.3 and C25.7–C25.9 | ﻿Sex, age, center, ﻿continuous variable and educational  level, physical activity, and smoking status as categorical variables ﻿with an additional category for missing values. |
| Fung,2010 | General population | SSB | | FFQ | Colorectal cancer | ﻿Sought  permission to obtain medical records to confirm the diagnosis. | ﻿Age, physical activity,  BMI, energy intake, alcohol intake, history of colorectal polyps, family history of colorectal cancer, history of  lower bowel endoscopy, aspirin use , packyears of smoking, and multivitamin use |
| Bao,2008 | General population | SSB; ASB; Total soft drinks | | FFQ | Pancreatic cancer | ﻿International Classification of  Diseases for Oncology, Third Edition: ICD-O-3 codes C25.0–  C25.3 and C25.7–C25.9 | ﻿Sex, race, education, BMI, alcohol (quintiles), physical activity, energy-adjusted red meat consumption, energy-adjusted folate intake(quintiles), andtotal energy, ﻿diet beverage consumption. |
| MICHAUD,1999 | General population | Fruit juice | | SFFQ | Bladder cancer | National Cancer Institute | Geographic region (five regions), age (in five-year categories), pack-years of smoking (six  categories), current smoking status (smoker or nonsmoker), energy intake (in quintiles), and intake of fruits and vegetables (five categories) |
| Nanclares, 2019 | General population | SSB | | SFFQ | Breast cancer | ﻿A trained  oncologist confirmed the cases. | ﻿Height, number of relatives with history of BC, smoking status, physical activity, alcohol intake, BMI, age of menarche, menopause  , number of pregnancies of more than 6 months, pregnancy before the age of 30 years, months of breastfeeding, use of hormone replacement  therapy and its duration and years at university, diabetes, glycemic index, total energy intake, decaffeinated coffee consumption, coffee consumption, ultra-processed food consumption and Mediterranean diet adherence |
| Chazelas,2019 | General population | SSB; ASB; Fruit juice | | 24-hour dietary records | Total cancer; Breast cancer; Colorectal cancer; Prostate cancer | ﻿ICD-10 | ﻿Age, sex, energy intake without alcohol, sugar intake from other dietary sources, alcohol, sodium,  lipid and fruit and vegetable intakes,  body mass index, height  , physical activity, smoking  s, number of 24 hour dietary records, family history of cancer educational, and the following prevalent conditions at baseline: type 2 diabetes, hypertension, major cardiovascular event, and dyslipidaemia We made additional adjustments for the number of biological children (continuous), menopausal status at baseline (menopausal or non-menopausal), hormonal  treatment for menopause at baseline and during follow-up (for postmenopausal analyses; yes or no), and oral contraception use at baseline and during follow-up (for premenopausal analyses; yes or no) for breast cancer analyses. |
| Stepien, 2014 | General population | Total soft drinks | | Country-specific dietary questionnaires | Hepatocellular carcinoma | ﻿ICD10 | ﻿Non-alcoholic energy intake  using the standard method, i.e. by adding to the model  (29)) and multivariable (additionally adjusted for a priori selected relevant confounders: smoking status and intensity of smoking (never; former smoker: quit <10 years ago, quit11–20 years ago, quit >20 years ago; Current smoker: 1–15 cigarettes/day, 16–25 cigarettes/day, >25 cigarettes/day; other than cigarettes; current/former missing; unknown), alcohol intake at recruitment (g/day, continuous) and lifetime pattern of alcohol intake (never, former light, former heavy, light, never heavy, periodically heavy, always  heavy drinkers, unknown); body mass index (BMI; kg/m2,  continuous), sex-specific physical activity (inactive, moderately active, active and missing), highest level of education attained (as a proxy for socio-economic status; none/  primary, technical/professional, secondary, university or  higher) and self-reported diabetes status (yes, no, missing)). |
| Wu, 2015 | General population | Fruit juice | | FFQ | Melanoma | ﻿We sought  permissionfromtheseparticipantstoacquiretheirmedicalandpathologicreports,  whichwerereviewedbystudyphysicianstovalidatethediagnoses. | ﻿age, family history of melanoma, natural hair color, No. of arm moles, sunburn susceptibility as child or adolescent, No.  of lifetime blistering sunburns, cumulative ultraviolet flux since baseline, average time spent in direct sunlight since high school, body mass index, physical activity,  smoking status, and intake of total energy, alcohol, coffee, vitamin C from supplements, and other individual citrus products (grapefruit juice, oranges, and orange  juice). Analyses for women were also adjusted for menopausal status and postmenopausal hormone use. |
| Ros, 2010 | General population | Total soft drink | | FFQ | All urothelial cell carcinomas | ﻿code C67 according to the ICD-Oncology | ﻿Age at entry, sex and centre and adjusted for smoking status (never, former and current), duration of smoking  (former and current smokers), lifetime intensity of smoking (former and current smokers), energy intake from fat and nonfat sources. |
| Allen, 2011 | General population | SSB; Fruit juice | | FFQ | Kidney cancer | ﻿ICD-10 | ﻿Age, region of residence, socioeconomic status, smoking,  and body mass index. |
| Khan, 2004 | General population | SSB | | FFQ | Total cancer; Colorectal cancer | ICD-9 | ﻿adjusted for age, health status,  health education, health screening & smoking |
| Friberg, 2011 | General population | Total soft drink | | FFQ | Endometrial cancer | ﻿Diabetes history was obtained through linkage of the  cohort to the Swedish In-patient Register, to the recent  National Diabetes Register, or self-reported on the second  questionnaire. | ﻿age in months, BMI (<20, 20–25, 26–30, >30 kg/m2), coffee (g/d,  continuous), energy (kcal/d, continuous), diabetes (yes/no), ﻿history of smoking (never/ever/missing). |
| Zhang, 2010 | General population | SSB | | FFQ | colon cancer | medical  records, linkage to cancer registries | Education; smoking; height; BMI; physical activity; family history of colorectal cancer; use of nonsteroidal anti-inflammatory  drugs; ultivitamin use; red meat intake; total milk consumption; alcohol; dietary folate intake; total energy intake; and in women, oral contraceptive use; and postmenopausal hormone use  . Age in years and questionnaire return year were included as stratification variables |
| Heath, 2021 | General population | Total soft drink; SSB; ASB; Fruit juice | | Diet questionnaires | Kidney cancer | linkage to population registries | Sex and country and adjusted for age at recruitment (years), educational attainment (none/primary school, technical or professional school, secondary  school, longer education including university), smoking status (never, former, current), alcohol consumption (continuous, g/day), physical activity (inactive, moderately inactive, moderately active, active),  juice intake (continuous, g/day; for soft drink analyses), and total soft drink intake (continuous, g/day; for juice analyses). Sugar-sweetened and artificially-sweetened soft drinks were mutually adjusted |
| Arthur, 2021 | General population | SSB; Fruit juice | | SFFQ | Breast, Endometrial, Ovarian, Colorectal cancer | Record  linkage to the National Mortality Database | Education, smoking, alcohol intake, physical activity, age at menarche, parity, age at menopause, HRT use, oral contraceptive  use, family history of breast cancer, history of benign breast disease, AHEI, BMI |
| Yuan, 2022 | General population | SSB | | FFQ | Overall, Colon, Rectum cancer | Self-report on biennial questionnaires or during follow-up  of participant deaths. | Sex; race (white, nonwhite, or unknown); family history of CRC (yes or no); BMI (in kg/m2) (<25.0, 25.0–29.9, 30.0–34.9, ≥35.0, or missing); physical activity (quintiles by sex); pack-years of  smoking (0, 1–4, 5–19, 20–39, ≥40, or missing); alcohol intake (g/d) (0, 0.1–4.9, 5.0–14.9, 15.0–29.9, or ≥30.0); regular aspirin use (yes or no); diabetes history (yes or no); lower endoscopy (never or ever); menopausal status and  hormone therapy use in women (premenopausal, postmenopausal never user, postmenopausal past user, postmenopausal current user, or unknown); intakes of dietary fiber, total calcium, total folate, red meat, and processed meat  (quintiles by sex); and Alternate Healthy Eating Index 2010 (without SSBs and fruit juice; quintiles by sex) |
| Hur, 2021 | General population | SSB; ASB, Fruit juice | | FFQ | Colorectal cancer | Medical records | race (white, non-white), height (continuous), body mass index at age 18 years (continuous), pack-years of smoking before age 20 years (continuous),  intake of alcohol at age 15–17 years, red and processed meat, dietary fibre, total folate (from foods and supplements) and total calcium at age 13–18 years (all continuous),  multivitamin use at age 13–18 years (yes, no) and physical activity at grade 9–12 (continuous) |
| Chen, 2021 | General population | SSB | | Questionnaire | Pancreatic cancer | National Cancer Registry | Age, gender, education levels, smoking status, drinking status, physical activity, body mass index, hypertension, and diabetes |
| Romanos-Nanclares, 2021 | General population | SSB; ASB | | FFQ | Breast cancer | Self-report on the  biennial questionnaires or National Death Index | Age in months and calendar year, adjusted for SSB or ASB intake, race, age at  menarche, postmenopausal hormone use, oral contraceptive use history, parity and age at first birth, breastfeeding history, family history of breast cancer,  history of benign breast disease, height, cumulatively updated alcohol intake,  cumulatively updated total caloric intake, physical activity, BMI at age 18 years, a  modified Alternate Healthy Eating Index score, and socioeconomic status |
| Ringel, 2022 | General population | ASB | | NR | Urinary tract cancer | NR | NR |

SSB: sugar sweetened beverages; ASB: artificially sweetened beverages; ICD: International Statistical Classification of Diseases; FFQ: food-frequency questionnaire; SFFQ: semiquantitative food-frequency questionnaire

**Table S5 Detailed guidance for assessment of risk of bias**

| **Item** | **Instruction** | |
| --- | --- | --- |
| Was selection of exposed and non-exposed cohorts drawn from the same population? | Definitely Yes | Studies in which selection for participation is not dependent on exposure level. For example, the European Prospective Investigation into Cancer and Nutrition study participants who were Recruited between January 1, 1992, and December 31,2000, predominantly from the general populations of 10European countries (Denmark, France, Germany, Greece, Italy, the Netherlands, Norway, Spain, Sweden, and the United Kingdom) |
|  | Probably Yes | / |
|  | Probably No | / |
|  | Definitely No | Studies that compare sugar-sweetened beverage and artificially sweetened beverage populations but draw sugar-sweetened beverage population from a different cohort. For example, a study may report on the EPIC-Oxford cohort but also include a subsample of participants from the Oxford Vegetarian Study. The study may then compare sugar-sweetened beverage population from the EPIC-Oxford cohort with artificially sweetened beverage populations from Oxford Vegetarian study. |
| Can we be confident in the assessment of exposure? | Definitely Yes | Participants complete sugar beverage measure at least once every five years. The sugar beverage measure (in most cases, this is a semi-quantitative food frequency questionnaire (FFQ)) has undergone validation against a weighted food record specifically for sugar beverage. |
|  | Probably Yes | Participants complete a dietary measure at least once every six to eight years. The dietary measure (in most cases, this is a semi-quantitative food frequency questionnaire (FFQ)) has undergone validation against a dietary measure other than a weighted food record (e.g., 24 h food record, biomarker). Some studies may provide a citation to the study validating the dietary measure and other studies may simply say that the measure has been validated against another dietary measure. |
|  | Probably No | Participants complete a dietary measure at least once every nine to 10 years. The dietary measure (in most cases, this is a semi-quantitative food frequency questionnaire (FFQ)) has not undergone any validation or the authors of the study do not report on the validity of the dietary measure. |
|  | Definitely No | Participants complete a dietary measure only at baseline. The dietary measure (in most cases, this is a semi-quantitative food frequency questionnaire (FFQ)) has not undergone any validation or the authors of the study do not report on the validity of the dietary measure.  Some studies may report that diet was assessed at multiple time points throughout the trial but only baseline dietary data is used for analysis. |
| Can we be confident that the outcome of interest was not present at start of study? | Definitely Yes | The outcome of interest is fatal. In that case, we can be certain that participants did not have the outcome at baseline. |
|  | Probably Yes | The authors have made an effort to exclude participants with the outcome of interest at baseline. The outcome is self-reported and there is no external validation. |
|  | Probably No | / |
|  | Definitely No | The authors have made no effort to exclude participants with the outcome of interest at baseline |
| Did the study match exposed and unexposed for all variables that are associated with the outcome of interest or did the statistical analysis adjust for these prognostic variables? | Definitely Yes | The study adjusts at a minimum for 1)age, 2) sex, 3) smoking at least one measure of 4) socioeconomic status such as level of income or education or occupation, 5) family history, 6) alcohol consumption, 7) weight or BMI and 8) physical activity in the analysis. |
|  | Probably Yes | Adjusts at a minimum for age, sex, smoking, family history, and weight or BMI |
|  | Probably No | Adjusts at a minimum for age, sex, and smoking |
|  | Definitely No | The study does not adjust for any prognostic variables relevant to the outcome or does not adjust for age, sex |
| Can we be confident in the assessment of the presence or absence of prognostic factors? | Definitely Yes | Typically, prognostic factors are self-reported by participants. This is considered acceptable. |
|  | Probably Yes | / |
|  | Probably No | Some studies may make assumptions regarding various prognostic factors. For example, a study may assume that all participants who did not answer the question on diabetes disease at baseline did not have diabetes |
|  | Definitely No | / |
| Can we be confident in the assessment of outcome? | Definitely Yes | All-cause mortality based on a government registry (e.g., National Death Index) with or without review by study physician or study staff  National or local registries (e.g., National Program of Cancer Registries (NPCR))with review by a study physician or study staff  Medical records reviewed by a study physician or study staff |
|  | Probably Yes | / |
|  | Probably No | Medical records without review by study physician or study staff |
|  | Definitely No | Report with no external validation |
| Was the follow-up of cohorts adequate? | Definitely Yes | At least 90% retention for the duration of the study. |
|  | Probably Yes | 80 to 89% retention for the duration of the study with loss to follow-up unlikely to be related to outcomes |
|  | Probably No | 80 to 89% retention for the duration of the study with loss to follow-up likely to be related to outcomes |
|  | Definitely No | Less than 80% follow-up. y |

NOTE: the criterion of guidance for assessment of risk of bias was cite from *Zeraatkar D, Han MA, Guyatt GH, et al. Red and Processed Meat Consumption and Risk for All-Cause Mortality and Cardiometabolic Outcomes: A Systematic Review and Meta-analysis of Cohort Studies. Ann Intern Med. 2019 19; 171(10):703-710. doi: 10.7326/M19-0655.*

# Table S6 Results of risk of bias assessment

| Author | Cohort  Name | Was selection of exposed and non-exposed cohorts drawn from the same population? | Can we be confident in the assessment of exposure? | Can we be confident that the outcome of interest was not present at start of study? | Did the study match exposed and unexposed for all variables that are associated with the outcome of interest or did the statistical analysis adjust for these prognostic variables? | Can we be confident in the assessment of the presence or absence of prognostic factors? | Can we be confident in the assessment of outcome? | Was the follow-up of cohorts adequate? | Overall |
| --- | --- | --- | --- | --- | --- | --- | --- | --- | --- |
| Bassett, 2019 | MCCS | DY | DN | PY | PY | DY | DY | PY | Low |
| Hodge, 2017 | MCCS | DY | DN | PY | PY | DY | DY | DN | High |
| Luo, 2019 | NHS | DY | DY | DY | DY | DY | DY | DY | Low |
| Luo, 2019 | HPFS | DY | DY | DY | DY | DY | DY | DY | Low |
| Schernhammer, 2012a | NHS | DY | DY | PY | DY | DY | DY | DN | Low |
| Schernhammer, 2012b; | HPFS | DY | DY | PY | DY | DY | DY | DY | Low |
| Lee,2006 | NHS | DY | DY | DY | DY | DY | DY | DY | Low |
| Lee,2006 | HPFS | DY | DY | DY | DY | DY | DY | DY | Low |
| Schernhammer,2005 | NHS | DY | DY | DY | DY | DY | DY | DY | Low |
| Schernhammer,2005 | HPFS | DY | DY | DY | DY | DY | DY | DY | Low |
| Wu, 2015a | NHS | DY | DY | PY | DY | DY | DY | PN | Low |
| Wu, 2015a | HPFS | DY | DY | PY | DY | DY | DY | PY | Low |
| Wu, 2015b | NHS/HPFS | DY | DY | PY | DY | DY | DY | DN | Low |
| Fung,2010 | NHS | DY | DY | PY | DY | DY | DY | DY | Low |
| Fung,2010 | HPFS | DY | DY | PY | DY | DY | DY | DY | Low |
| MICHAUD, 1999 | HPFS | DY | DY | DY | DY | DY | DY | DY | Low |
| Larsson, 2016 | SMC | DY | DN | DY | DY | DY | DY | PN | High |
| Larsson, 2016 | COSM | DY | DN | DY | DY | DY | DY | PY | Low |
| Larsson, 2006 | SMC | DY | DN | DY | DY | DY | DY | DN | High |
| Larsson, 2006 | COSM | DY | DN | DY | DY | DY | DY | DN | High |
| Pacheco, 2019 | CTS | DY | DN | PN | DY | DY | DY | DN | High |
| Miles, 2018 | PLCO | DY | DN | PN | PY | DY | DY | DY | High |
| Navarrete-Muñoz,2016 | EPIC | DY | DN | PY | DY | DY | PY | DY | Low |
| Stepien, 2014 | EPIC | DY | DN | DY | DY | DY | DY | PY | Low |
| Ros, 2010 | EPIC | DY | DN | PY | DY | DY | DY | DN | High |
| Zamora-Ros, 2017 | EPIC | DY | DN | PY | DY | DY | DY | DY | Low |
| Mueller,2010 | SCH | DY | DN | DY | DY | DY | DY | DY | Low |
| Odegaard, 2014 | SCH | DY | DN | DY | DY | DY | DY | DY | Low |
| McCullough, 2014; | CPS-II Nutrition Cohort | DY | DN | DY | DY | DY | DY | DY | Low |
| Choi, 2013; | IWHS | DY | DN | DY | DY | DY | DY | DY | Low |
| Drake, 2012; | MDC | DY | DN | DY | DY | DY | DY | DY | Low |
| Ren, 2010 | NIH-AARP Diet and Health Study | DY | PN | PY | DY | DY | DY | DY | Low |
| Bao,2008 | NIH-AARP Diet and Health Study | DY | PN | PY | DY | DY | DY | DY | Low |
| Nöthlings, 2007 | Multiethnic Cohort Study | DY | DN | PN | DY | DY | DY | DY | High |
| Ellison,2000; | NCS | DY | DN | PY | PN | DY | DY | DN | High |
| Debras,2020 | NutriNet-Santé cohort | DY | DY | PY | DY | DY | DY | DY | Low |
| Chazelas,2019 | NutriNet-Santé cohort | DY | DY | PY | DY | DY | DY | DY | Low |
| Makarem, 2018; | FOS | DY | DY | PY | DY | DY | DY | DY | Low |
| Nanclares, 2019; | SUN | DY | DN | PY | DY | DY | DY | DY | Low |
| Allen, 2011; | The Million Women Study | DY | DY | PY | PN | DY | DY | PY | Low |
| Friberg, 2011 | Swedish Mammography Cohort, | DY | DN | PY | PY | DY | DY | DN | High |
| Khan, 2004 | Hokkaido | DY | DN | PY | PY | DY | DY | PY | Low |
| Zhang, 2010 | Pooled Analysis | DY | DN | PY | PY | DY | DY | PY | Low |
| Heath, 2021 | EPIC | DY | DN | PY | DY | DY | DY | DY | Low |
| Arthur, 2021 | CSDLH | DY | DN | PY | PY | DY | DY | DY | Low |
| Yuan, 2022 | HPFS | DY | DY | PY | DY | DY | DY | DY | Low |
| Yuan, 2022 | NHS | DY | DY | DY | DY | DY | DY | DY | Low |
| Hur, 2021 | NHSII | DY | DY | DY | DY | DY | DY | DY | Low |
| Chen, 2021 | HMAC | DY | DN | PY | PY | DY | DY | PY | Low |
| Romanos-Nanclares, 2021 | NHS | DY | DY | DY | DY | DY | DY | DY | Low |
| Romanos-Nanclares, 2021 | NHSII | DY | DY | DY | DY | DY | DY | DY | Low |
| Ringel, 2022 | NR | DY | DY | PY | PY | DY | PY | PY | Low |

DY: Definitely Yes; PY: Probably Yes; DN: Definitely No; PN: Probably No; EPIC: European Prospective Investigation into Cancer and Nutrition; NHS: Nurses’ Health Study; SCHS: Singapore Chinese Health Study; CPS: Cancer Prevention Study; IWHS: Iowa Women’s Health Study; MDC: Malmo¨ Diet and Cancer; SMC: Swedish Mammography Cohort; COSM: Cohort of Swedish Men; MCCS: Melbourne Collaborative Cohort Study; CTS: California Teachers Study; PLCO: Prostate, Lung, Colorectal, and Ovarian; NCS: Nutrition Canada Survey; FOS: The Framingham Offspring; SUN: Seguimiento Universidad de Navarra

# Table S7 Summary of findings for beverages consumption (per 250mL/day increase) and specific cancer risk

| **Outcomes** | **Studies, n** | **Mean Follow-up, y** | **RR (95%CI)** | **Population risk per 1000 persons over 10.8 y*** | **Risk difference**  **per 1000 person (95%CI)** | **GRADE certainty of evidence** | **Summary** | | |
| --- | --- | --- | --- | --- | --- | --- | --- | --- | --- |
| **SSBs** | | | | | | | |  |  |
| Overall cancer | 4 | 13.00 | 1.07 (0.95 to 1.22) | 185 | 13 (-9 to 41) | Very low$¶ | We are uncertain of the effects of Per 250mL/day increase of SSBs consumption and all cancer risk | | |
| Breast cancer | 7 | 8.15 | 1.17 (1.00 to 1.37) | 46 | 8 (0 to 17) | Moderate $# | Per 250mL/day increase of SSBs consumption is likely to have small effect on breast cancer risk | | |
| Colorectal cancer | 8 | 15.68 | 1.10 (1.04 to 1.15) | 20 | 2 (1 to 3) | Moderate$# | Per 250mL/day increase of SSBs consumption is likely to have small effect on colorectal cancer risk | | |
| Endometrial cancer | 2 | 7.80 | 1.01 (0.99 to 1.03) | 10 | 0 (0 to 0) | Very low$¶ | We are uncertain of the effects of Per 250mL/day increase of SSBs consumption and endometrial cancer risk | | |
| Esophagus cancer | 1 |  | 0.91 (0.74 to 1.11) | 7 | -1 (-2 to 1) | Very low$¶ | We are uncertain of the effects of Per 250mL/day increase of SSBs consumption and esophagus cancer risk | | |
| Biliary tract cancer | 1 | 13.4 | 1.30 (1.10 to 1.54) | 2 | 1 (0 to 1) | Low$ | Per 250mL/day increase of SSBs consumption is likely to have small effect on Biliary tract cancer risk | | |
| Gastric cancer | 2 | 11,6 | 1.00 (0.85 to 1.17) | 14 | 0 (-2 to 2) | Very low$¶ | We are uncertain of the effects of Per 250mL/day increase of SSBs consumption and Gastric cancer risk | | |
| Hepatocellular carcinoma | 1 | 32 | 1.06 (0.95 to 1.18) | 11 | 1 (-1 to 2) | Very low$¶ | We are uncertain of the effects of Per 250mL/day increase of SSBs consumption and hepatocellular carcinoma risk | | |
| Kidney cancer | 3 | 12.7 | 1.06 (0.98 to 1.15) | 5 | 0 (0 to 1) | Low$¶# | We are uncertain of the effects of Per 250mL/day increase of SSBs consumption and kidney cancer risk | | |
| Leukemia | 2 | 22 | 1.06 (0.73 to 1.54) | 4 | 0 (-1 to 2) | Very low$¶ | We are uncertain of the effects of Per 250mL/day increase of SSBs consumption and Leukemia risk | | |
| Multiple myeloma | 2 | 22 | 1.18 (0.90 to 1.55) | 2 | 0 (0 to 1) | Very low$¶ | We are uncertain of the effects of Per 250mL/day increase of SSBs consumption and Multiple myeloma risk | | |
| Non-Hodgkin lymphoma | 3 | 16 | 1.07 (0.92 to 1.23) | 5 | 0 (0 to 1) | Very low$¶ | We are uncertain of the effects of Per 250mL/day increase of SSBs consumption and Non-Hodgkin lymphoma risk | | |
| Oral cancer | 1 |  | 1.01 (0.83 to 1.23) | 5 | 0 (-1 to 1) | Very low$¶ | We are uncertain of the effects of Per 250mL/day increase of SSBs consumption and Oral cancer risk | | |
| Ovarian cancer | 1 | 11.6 | 1.22 (0.98 to 1.52) | 7 | 2 (0 to 4) | Very low$¶ | We are uncertain of the effects of Per 250mL/day increase of SSBs consumption and Ovarian cancer risk | | |
| Pancreatic cancer | 7 | 11.33 | 1.08 (0.97 to 1.21) | 5 | 0 (-1 to 2) | Low$¶ | We are uncertain of the effects of Per 250mL/day increase of SSBs consumption and pancreatic cancer risk | | |
| Pharynx cancer | 1 |  | 0.95 (0.72, 1.25) | 2 | 0 (-1 to 1) | Very low$¶ | We are uncertain of the effects of Per 250mL/day increase of SSBs consumption and Pharynx cancer risk | | |
| Larynx cancer | 1 |  | 0.97 (0.78, 1.21) | 3 | 0 (-1 to 1) | Very low$¶ | We are uncertain of the effects of Per 250mL/day increase of SSBs consumption and Larynx cancer risk | | |
| Prostate cancer | 5 | 8.57 | 1.10 (1.00 to 1.22) | 38 | 4 (0 to 8) | Low$ | Per 250mL/day increase of SSBs consumption is likely to have small effect on prostate cancer risk | | |
| **ASBs** | | | | | | | | |  |
| Overall cancer | 2 | 8.35 | 0.96 (0.86 to 1.08) | 185 | -7 (-26 to 15) | Very low$¶ | We are uncertain of the effects of Per 250mL/day increase of SSBs consumption and all cancer risk | | |
| Breast cancer | 3 | 8.35 | 0.95 (0.80 to 1.12) | 46 | -1 (-2 to 0) | Very low$¶ | We are uncertain of the effects of Per 250mL/day increase of SSBs consumption and breast cancer risk | | |
| Colorectal cancer | 2 | 11.6 | 0.93 (0.78 to 1.10) | 20 | -2 (-4 to 0) | Very low$¶ | We are uncertain of the effects of Per 250mL/day increase of SSBs consumption and Colorectal cancer risk | | |
| endometrial cancer | 1 | 11.6 | 0.93 (0.62 to 1.40) | 10 | -1 (-4 to 4) | Very low$¶ | We are uncertain of the effects of Per 250mL/day increase of SSBs consumption and endometrial cancer risk | | |
| Gastric cancer | 1 | 11.6 | 1.15 (0.76 to 1.74) | 14 | 2 (-3 to 10) | Very low$¶ | We are uncertain of the effects of Per 250mL/day increase of SSBs consumption and Gastric cancer risk | | |
| Kidney cancer | 1 | 11.6 | 0.88 (0.57 to 1.36) | 5 | -1 (-2 to 2) | Very low$¶ | We are uncertain of the effects of Per 250mL/day increase of SSBs consumption and kidney cancer risk | | |
| Leukemia | 1 | 22 | 1.16 (1.00 to 1.35) | 4 | 1 (0 to 1) | Low$ | Per 250mL/day increase of SSBs consumption is likely to have very small effect on prostate cancer risk | | |
| Multiple myeloma | 2 | 22 | 1.14 (0.81 to 1.60) | 2 | 0 (0 to 1) | Very low$¶ | We are uncertain of the effects of Per 250mL/day increase of SSBs consumption and multiple myeloma risk | | |
| Non-Hodgkin lymphoma | 3 | 16 | 1.00 (0.90 to 1.11) | 5 | 0 (-1 to 1) | Very low$¶ | We are uncertain of the effects of Per 250mL/day increase of SSBs consumption and non-Hodgkin lymphoma risk | | |
| Ovarian cancer | 1 | 11.6 | 1.31 (0.87 to 1.98) | 7 | 2 (-1 to 7) | Very low$¶ | We are uncertain of the effects of Per 250mL/day increase of SSBs consumption and Ovarian cancer risk | | |
| pancreatic cancer | 3 | 12.93 | 1.03 (0.96 to 1.10) | 5 | 0 (0 to 1) | Low$¶# | We are uncertain of the effects of Per 250mL/day increase of SSBs consumption and pancreatic cancer risk | | |
| Prostate cancer | 2 | 8.35 | 0.93 (0.69 to 1.26) | 38 | -3 (-12 to 10) | Very low$¶ | We are uncertain of the effects of Per 250mL/day increase of SSBs consumption and Prostate cancer risk | | |
| **100% Fruit juice** | | | | | | | |  |  |
| Overall cancer | 2 | 10.7 | 1.31 (1.04 to 1.65) | 185 | 57 (7 to 120) | Low$ | Per 250mL/day increase of SSBs consumption is likely to have small effect on all cancer risk | | |
| breast cancer | 3 | 5.1 | 1.07 (0.96 to 1.18) | 46 | 3 (-2 to 8) | Very low$¶ | We are uncertain of the effects of Per 250mL/day increase of SSBs consumption and breast cancer risk | | |
| basal cell carcinoma | 1 | 24-26 | 1.00 (0.96 to 1.04) |  |  |  |  | | |
| Colorectal cancer | 3 | 5.1 | 1.21 (1.00 to 1.47) | 20 | 4 (0 to 9) | Very low$¶ | We are uncertain of the effects of Per 250mL/day increase of SSBs consumption and Colorectal cancer risk | | |
| endometrial cancer | 2 | 5 | 1.05 (1.00 to 1.10) | 10 | 1 (0 to 1) | Very low$¶ | We are uncertain of the effects of Per 250mL/day increase of SSBs consumption and endometrial cancer risk | | |
| Melanoma | 1 |  | 1.22 (1.14 to 1.31) | 3 | 1 (0 to 1) | Low$ | Per 250mL/day increase of SSBs consumption is likely to have small effect on Melanoma risk | | |
| pancreatic cancer | 3 | 11.2 | 0.91 (0.61 to 1.35) | 5 | 0 (-2 to 2) | Very low$¶# | We are uncertain of the effects of Per 250mL/day increase of SSBs consumption and pancreatic cancer risk | | |
| Prostate cancer | 3 | 8.57 | 1.13 (0.93 to 1.39) | 38 | 5 (-3 to 15) | Very low$¶ | We are uncertain of the effects of Per 250mL/day increase of SSBs consumption and Prostate cancer risk | | |
| squamous cell carcinoma | 1 | 24-26 | 1.02 (1.00 to 1.04) | 2 | 0 (0 to 0) | Low$ | We are uncertain of the effects of Per 250mL/day increase of SSBs consumption and squamous cell carcinoma risk | | |
| Thyroid cancer | 1 | 14 | 1.29 (1.08 to 1.53) | 4 | 1 (0 to 2) | Low$ | Per 250mL/day increase of SSBs consumption is likely to have very small effect on Thyroid cancer risk | | |
| **Total soft drink** | | | | | | | |  |  |
| Pancreatic cancer | 2 | 9.4 | 1.00 (0.95 to, 1.05) | 5 | 0 (0 to 0) | Very low$¶ | We are uncertain of the effects of Per 250mL/day increase of SSBs consumption and Pancreatic cancer risk | | |
| Urothelial cell carcinomas ^&^ | 1 | 9.3 | 1.08 (0.74 to 1.57) |  |  |  |  | | |
| Hepatocellular carcinoma | 1 | 11.4 | 1.42 (1.08 to 1.87) | 11 | 5 (1 to 9) | Low$ | Per 250mL/day increase of SSBs consumption is likely to have small effect on Thyroid cancer risk | | |

SSB: sugar-sweetened beverages; ASB: artificially sweetened beverages; RR: relative risk

GRADE: Grading of Recommendations Assessment, Development and Evaluation. CVD: cardiovascular disease. SSBs: Sugar-sweetened beverages. ASBs: Artificially sweetened beverages. HR: Hazard ratios.

* Population risk of cancer incidence comes from Lifetime cumulative risk from GLOBOCAN 2012.

& Population risk of cancer incidence comes from the cancer incidence of reference group.

$ Certainty of evidence starts from low due to observational design.

# Upgraded one level as dose-response gradient is present

¶ Downgraded one level for imprecision as confidence interval around absolute effect includes both small benefit and small harm.

⁑ Downgraded one level for risk of bias as subgroup analysis shows significant differences between low- and high-risk of bias.

# Table S8 Summary results of dose-response meta-analyses, subgroup analyses (increase 250ml per day)

| Subgroup factors | SSBs | | ASBs | | 100% fruit juices | |
| --- | --- | --- | --- | --- | --- | --- |
|  | RR (95%CI) | P _interaction_ | RR (95%CI) | P _interaction_ | RR (95%CI) | P _interaction_ |
| **Sex** |  |  |  |  |  |  |
| Men | / | / | / | / | / | / |
| Women | / |  | / |  | / |  |
| **Age** |  |  |  |  |  |  |
| **Endometrial cancer** |  |  |  |  |  |  |
| ≥60 | 1.01 (0.99, 1.03) | 0.92 | / | / | 1.05 (0.94, 1.18) | 0.69 |
| ﹤60 | 1.03 (0.69, 1.54) |  | / |  | 0.99 (0.76, 1.28) |  |
| **Non-Hodgkin lymphoma** |  |  |  |  |  |  |
| ≥60 | 1.03 (0.89, 1.19) | 0.73 | 0.91 (0.82, 1.00) | 0.05 | 1.03 (0.95, 1.11) | 0.86 |
| ﹤60 | 1.08 (0.84, 1.40) |  | 1.05 (0.95, 1.15) |  | 1.05 (0.79, 1.40) |  |
| **Pancreatic cancer** |  |  |  |  |  |  |
| ≥60 | 1.06 (0.81, 1.38) | 0.56 | 1.03 (0.95, 1.11) | 0.86 | 0.67 (0.39, 1.15) | 0.10 |
| ﹤60 | 1.24 (0.79, 1.96) |  | 1.05 (0.79, 1.40) |  | 1.33 (0.73, 2.43) |  |
| **Follow-up duration** |  |  |  |  |  |  |
| **Pancreatic cancer** |  |  |  |  |  |  |
| ≥10 years | 1.24 (0.79, 1.96) | 0.56 | 1.03 (0.95, 1.11) | 0.85 | 1.22 (0.62, 2.39) | 0.65 |
| <10 years | 1.06 (0.81, 1.38) |  | 1.01 (0.82, 1.24) |  | 0.93 (0.36, 2.40) |  |
| **Breast cancer** |  |  |  |  |  |  |
| ≥10 years | 1.15 (0.97, 1.35) | 0.12 | / | / | 1.33 (0.90, 1.97) | 0.40 |
| <10 years | 1.39 (1.17, 1.66) |  | / | / | 1.04 (0.69, 1.57) |  |
| **Prostate cancer** |  |  |  |  |  |  |
| ≥10 years | 1.10 (0.94, 1.28) | 0.63 |  |  | 1.18 (0.66, 2.10) | 0.99 |
| <10 years | 1.21 (0.83, 1.76) |  |  |  | 1.18 (0.80, 1.76) |  |
| **Overall cancer** |  |  |  |  |  |  |
| ≥10 years | 1.03 (0.92, 1.15) | 0.03 | / | / | 1.36 (1.08, 1.71) | 0.22 |
| <10 years | 1.36 (1.09, 1.70) |  | / |  | 0.99 (0.63, 1.55) |  |
| **Colorectal cancer** |  |  |  |  |  |  |
| ≥10 years | 1.10 (1.03, 1.16) | 0.65 | / | / | / | / |
| <10 years | 1.53 (0.36, 6.48) |  | / |  | / |  |
| **Kidney cancer** |  |  |  |  | / | / |
| ≥10 years | 1.11 (0.92, 1.35) | 0.60 | / | / | / |  |
| <10 years | 1.05 (0.96, 1.15) |  | / | / | / |  |
| **Risk of bias** |  |  |  |  |  |  |
| **Breast cancer** |  |  |  |  |  |  |
| Low risk | 1.39 (1.16, 1.65) | 0.11 | / | / | / | / |
| High risk | 1.14 (0.96, 1.35) |  | / |  | / |  |
| **Colorectal cancer** |  |  |  |  |  |  |
| Low risk | 1.09 (1.00, 1.18) | 0.74 | / | / | / | / |
| High risk | 1.11 (1.02, 1.21) |  | / | / | / | / |
| **Endometrial cancer** |  |  |  |  |  | / |
| Low risk | 1.01 (0.99, 1.03) | 0.92 | / |  |  | / |
| High risk | 1.03 (0.69, 1.54) |  | / |  | / | / |
| **Gastric cancer** |  |  |  |  | / | / |
| Low risk | 0.96 (0.80, 1.16) | 0.46 | / |  |  | / |
| High risk | 1.10 (0.81, 1.50) |  | / |  | / | / |
| **Kidney cancer** |  |  | / |  |  | / |
| Low risk | 1.05 (0.97, 1.14) | 0.32 | / |  | / | / |
| High risk | 1.26 (0.89, 1.79) |  | / |  | / | / |
| **Pancreatic cancer** |  |  |  |  |  | / |
| Low risk | 1.14 (0.93, 1.41) | 0.75 | / |  | 0.93 (0.36, 2.40) | 0.65 |
| High risk | 1.09 (0.90, 1.32) |  | / |  | 1.22 (0.62, 2.39) |  |
| **Overall cancer** |  |  | / |  |  |  |
| Low risk | 1.06 (0.88, 1.27) | 0.54 | / |  | / | / |
| High risk | 1.13 (1.03, 1.24) |  | / |  | / | / |
| **Study location** |  |  |  |  |  | **/** |
| **Breast cancer** |  |  |  |  | / | / |
| USA | 1.11 (0.23, 5.40) | 0.87 | / |  | 1.04 (0.69, 1.57) | 0.40 |
| Asia | / |  | / |  | / | / |
| Europe | 1.27 (1.08, 1.49) |  | / |  | 1.33 (0.90, 1.97) | / |
| **Colorectal cancer** |  |  | / |  |  | / |
| USA | 1.08 (1.01, 1.16) | 0.42 | / |  | / | / |
| Asia |  |  | / |  | / | / |
| Europe | 1.15 (1.01, 1.32) |  | / |  | / | / |
| **Endometrial cancer** |  |  | / |  |  |  |
| USA | 1.01 (0.99, 1.03) | 0.92 | / |  | / | / |
| Asia |  |  | / |  | / | / |
| Europe | 1.03 (0.69, 1.54) |  | / |  | / | / |
| **Kidney cancer** |  |  | / |  | / | / |
| USA | 1.05 (0.83, 1.33) | 0.93 | / | / | / | / |
| Asia |  |  | / | / | / | / |
| Europe | 1.06 (0.97, 1.16) |  | / | / | / | / |
| **Pancreatic cancer** |  |  | / | / | / | / |
| USA | 0.96 (0.87, 1.07) | 0.006 | 1.03 (0.96, 1.11) | 0.65 | 1.22 (0.62, 2.39) | 0.21 |
| Asia | 3.99 (1.11, 14.36) |  | / | / | 1.90 (0.49, 7.38) |  |
| Europe | 1.22 (1.04, 1.43) |  | 0.96 (0.71, 1.30) | / | 0.67 (0.39, 1.15) |  |
| **Prostate cancer** |  |  |  | / |  |  |
| USA | 1.00 (0.68, 1.48) | 0.56 | / | / | 1.49 (1.02, 2.18) | 0.10 |
| Asia |  |  | / | / |  |  |
| Europe | 1.13 (0.97, 1.31) |  | / | / | 1.02 (0.80, 1.29) |  |
| **Overall cancer** |  |  |  | / |  |  |
| USA |  | 0.01 | / | / |  | 0.22 |
| Asia | 0.89 (0.77, 1.03) |  | / | / | 0.99 (0.63, 1.55) |  |
| Europe | 1.13 (1.01, 1.26) |  | / | / | 1.36 (1.08, 1.71) |  |

SSB: sugar-sweetened beverages; ASB: artificially sweetened beverages; RR: risk ratio; we did not perform subgroup analysis for the cancer types that were not listed in our table because of limited study number; /: we did not perform subgroup analysis because of limited study number


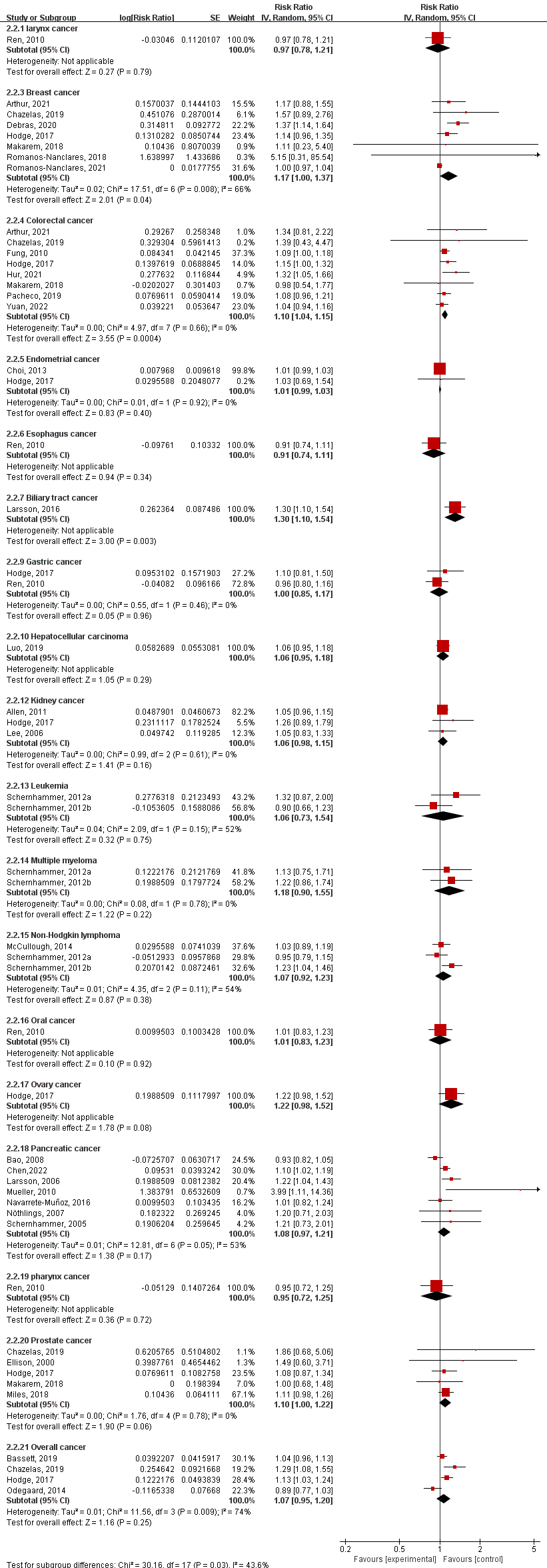


# Figure S1 Results of Sugar-sweetened beverages consumption and the risk of cancer incidence in highest versus lowest comparison


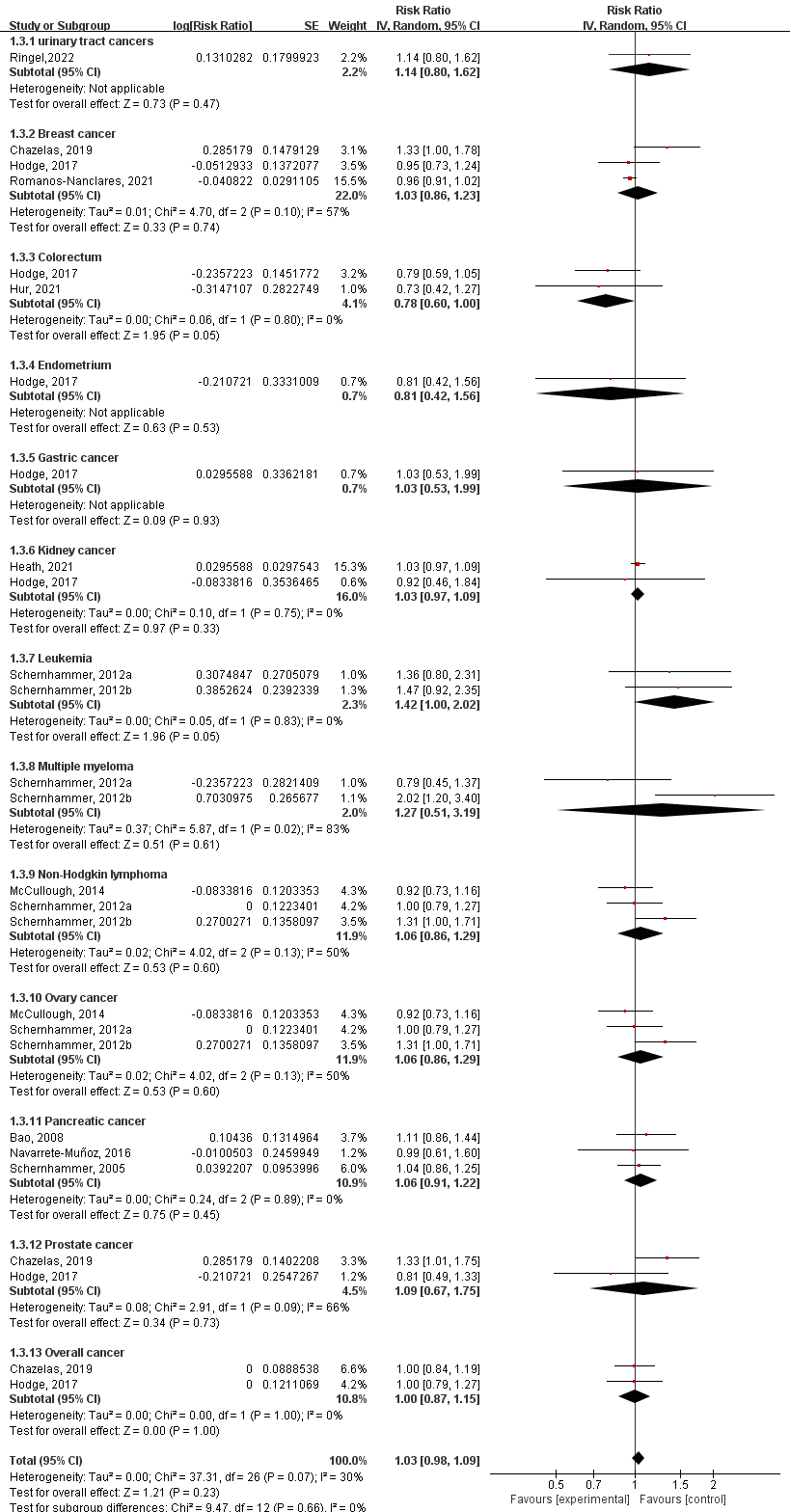


# Figure S2 Results of Artificially sweetened beverages consumption and the risk of cancer incidence in highest versus lowest comparison


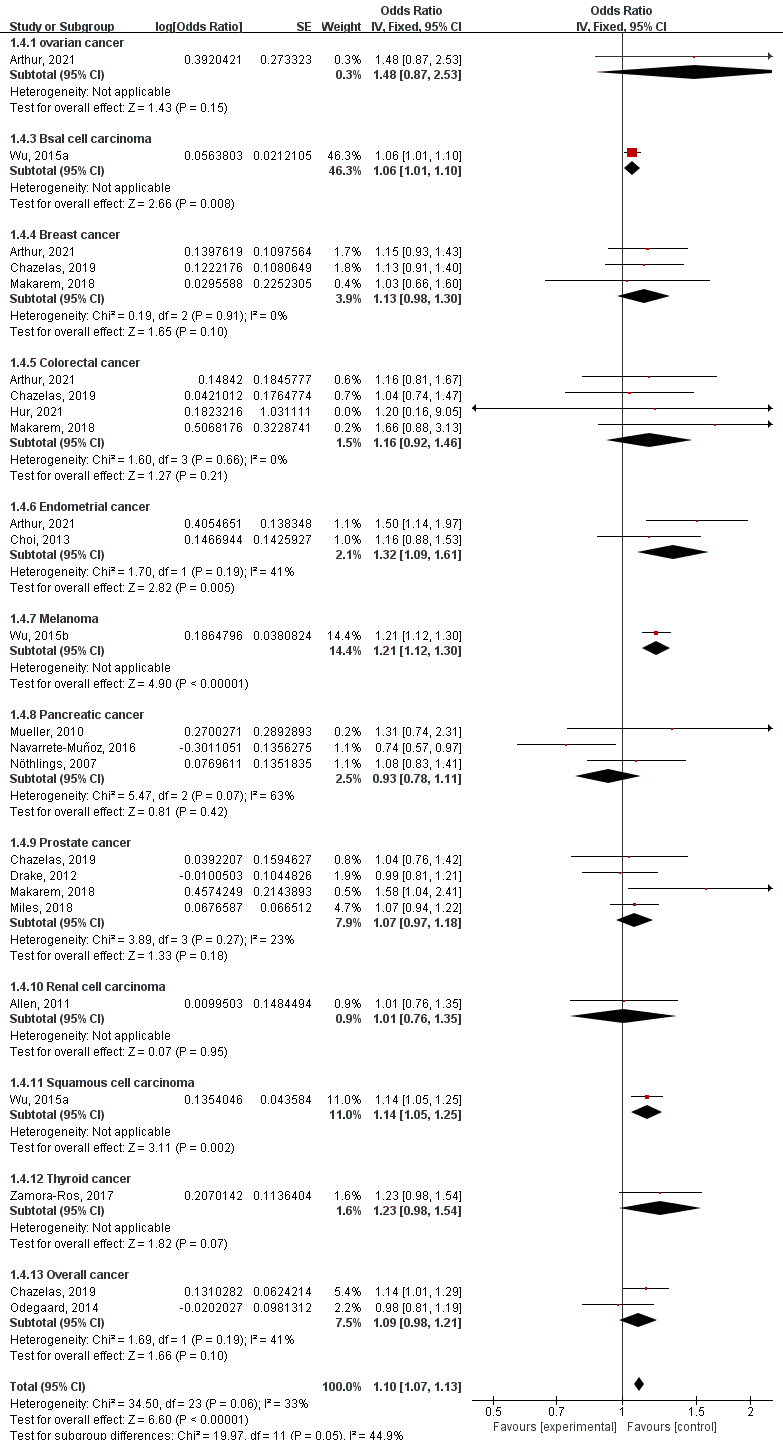


# Figure S3 Results of 100% Fruit Juices consumption and the risk of cancer incidence in highest versus lowest comparison


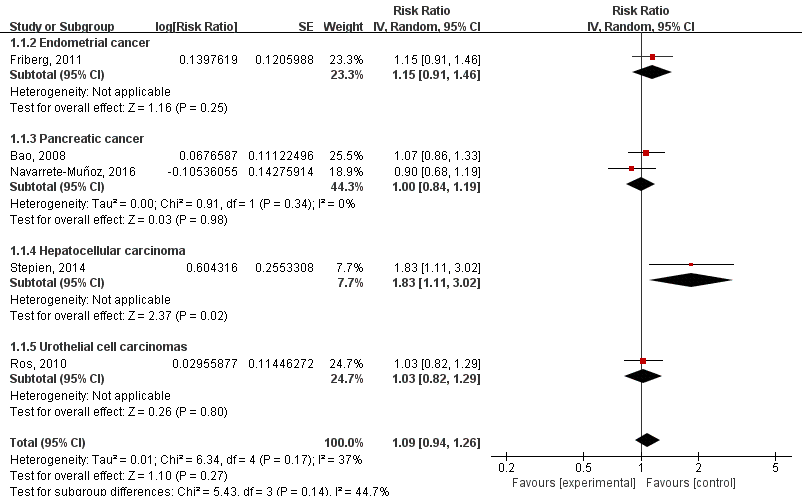


# Figure S4 Results of Total soft drinks consumption and the risk of cancer incidence in never versus ever comparison

# Appendix 1 the lists of studies excluded in full-text screen

**References:**

1. Fung TT, Kashambwa R, Sato K, Chiuve SE, Fuchs CS, Wu K, Giovannucci E, Ogino S, Hu FB, Meyerhardt JA. Post diagnosis diet quality and colorectal cancer survival in women. PLoS One. 2014 Dec 15;9(12):e115377. doi: 10.1371/journal.pone.0115377. PMID: 25506700; PMCID: PMC4266679.
2. Mullee A, Romaguera D, Pearson-Stuttard J, Viallon V, Stepien M, Freisling H, Fagherazzi G, Mancini FR, Boutron-Ruault MC, Kühn T, Kaaks R, Boeing H, Aleksandrova K, Tjønneland A, Halkjær J, Overvad K, Weiderpass E, Skeie G, Parr CL, Quirós JR, Agudo A, Sánchez MJ, Amiano P, Cirera L, Ardanaz E, Khaw KT, Tong TYN, Schmidt JA, Trichopoulou A, Martimianaki G, Karakatsani A, Palli D, Agnoli C, Tumino R, Sacerdote C, Panico S, Bueno-de-Mesquita B, Verschuren WMM, Boer JMA, Vermeulen R, Ramne S, Sonestedt E, van Guelpen B, Holgersson PL, Tsilidis KK, Heath AK, Muller D, Riboli E, Gunter MJ, Murphy N. Association Between Soft Drink Consumption and Mortality in 10 European Countries. JAMA Intern Med. 2019 Sep 3:e192478. doi: 10.1001/jamainternmed.2019.2478. Epub ahead of print. Erratum in: JAMA Intern Med. 2019 Nov 1;179(11):1607. PMID: 31479109; PMCID: PMC6724165.
3. Haridass V, Ziogas A, Neuhausen SL, Anton-Culver H, Odegaard AO. Diet Quality Scores Inversely Associated with Postmenopausal Breast Cancer Risk Are Not Associated with Premenopausal Breast Cancer Risk in the California Teachers Study. J Nutr. 2018 Nov 1;148(11):1830-1837. doi: 10.1093/jn/nxy187. PMID: 30247577.
4. Sharma I, Roebothan B, Zhu Y, Woodrow J, Parfrey PS, Mclaughlin JR, Wang PP. Hypothesis and data-driven dietary patterns and colorectal Cancer survival: findings from Newfoundland and Labrador colorectal Cancer cohort. Nutr J. 2018 May 25;17(1):55. doi: 10.1186/s12937-018-0362-x. PMID: 29793493; PMCID: PMC5968482.
5. Fuchs MA, Sato K, Niedzwiecki D, Ye X, Saltz LB, Mayer RJ, Mowat RB, Whittom R, Hantel A, Benson A, Atienza D, Messino M, Kindler H, Venook A, Ogino S, Wu K, Willett WC, Giovannucci EL, Meyerhardt JA. Sugar-sweetened beverage intake and cancer recurrence and survival in CALGB 89803 (Alliance). PLoS One. 2014 Jun 17;9(6):e99816. doi: 10.1371/journal.pone.0099816. PMID: 24937507; PMCID: PMC4061031.
6. Ramne S, Alves Dias J, González-Padilla E, Olsson K, Lindahl B, Engström G, Ericson U, Johansson I, Sonestedt E. Association between added sugar intake and mortality is nonlinear and dependent on sugar source in 2 Swedish population-based prospective cohorts. Am J Clin Nutr. 2019 Feb 1;109(2):411-423. doi: 10.1093/ajcn/nqy268. PMID: 30590448.
7. Braverman-Bronstein A, Camacho-García-Formentí D, Zepeda-Tello R, Cudhea F, Singh GM, Mozaffarian D, Barrientos-Gutierrez T. Mortality attributable to sugar sweetened beverages consumption in Mexico: an update. Int J Obes (Lond). 2020 Jun;44(6):1341-1349. doi: 10.1038/s41366-019-0506-x. Epub 2019 Dec 10. PMID: 31822805.
8. Ross SM. Sugary Drinks: A Modifiable Risk Factor for Cancer Prevention. Holist Nurs Pract. 2019 Nov/Dec;33(6):366-369. doi: 10.1097/HNP.0000000000000358. PMID: 31609874.
9. Fassier P, Zelek L, Lécuyer L, Bachmann P, Touillaud M, Druesne-Pecollo N, Galan P, Cohen P, Hoarau H, Latino-Martel P, Kesse-Guyot E, Baudry J, Hercberg S, Deschasaux M, Touvier M. Modifications in dietary and alcohol intakes between before and after cancer diagnosis: Results from the prospective population-based NutriNet-Santé cohort. Int J Cancer. 2017 Aug 1;141(3):457-470. doi: 10.1002/ijc.30704. Epub 2017 May 15. PMID: 28335085; PMCID: PMC5655904.
10. Miles FL, Chang SC, Morgenstern H, Tashkin D, Rao JY, Cozen W, Mack T, Lu QY, Zhang ZF. Association of sugary beverages with survival among patients with cancers of the upper aerodigestive tract. Cancer Causes Control. 2016 Nov;27(11):1293-1300. doi: 10.1007/s10552-016-0792-8. Epub 2016 Aug 18. Erratum in: Cancer Causes Control. 2016 Nov;27(11):1301. PMID: 27539643; PMCID: PMC5552047.
11. Moreano B. Gallenblasenkrebs durch Limonade? (Gallbladder cancer caused by soft drinks?). MMW Fortschr Med. 2016 Jun 23;158(12):3. German. PMID: 27462659.
12. Akinyemiju T, Moore JX, Pisu M, Lakoski SG, Shikany J, Goodman M, Judd SE. A prospective study of dietary patterns and cancer mortality among Blacks and Whites in the REGARDS cohort. Int J Cancer. 2016 Nov 15;139(10):2221-31. doi: 10.1002/ijc.30287. Epub 2016 Aug 9. PMID: 27459634; PMCID: PMC5041524.
13. La Vecchia C. Re: consumption of artificial sweetener- and sugar-containing soda and the risk of lymphoma and leukemia in men and women. Am J Clin Nutr. 2013 May;97(5):1153. doi: 10.3945/ajcn.112.055640. PMID: 23604437.
14. Aune D. Soft drinks, aspartame, and the risk of cancer and cardiovascular disease. Am J Clin Nutr. 2012 Dec;96(6):1249-51. doi: 10.3945/ajcn.112.051417. Epub 2012 Nov 7. PMID: 23134894; PMCID: PMC3497921.
15. Genkinger JM, Li R, Spiegelman D, Anderson KE, Albanes D, Bergkvist L, Bernstein L, Black A, van den Brandt PA, English DR, Freudenheim JL, Fuchs CS, Giles GG, Giovannucci E, Goldbohm RA, Horn-Ross PL, Jacobs EJ, Koushik A, Männistö S, Marshall JR, Miller AB, Patel AV, Robien K, Rohan TE, Schairer C, Stolzenberg-Solomon R, Wolk A, Ziegler RG, Smith-Warner SA. Coffee, tea, and sugar-sweetened carbonated soft drink intake and pancreatic cancer risk: a pooled analysis of 14 cohort studies. Cancer Epidemiol Biomarkers Prev. 2012 Feb;21(2):305-18. doi: 10.1158/1055-9965.EPI-11-0945-T. Epub 2011 Dec 22. PMID: 22194529; PMCID: PMC3275675.
16. Thomson CA, Martínez ME. Coffee, tea, what beverage for me? Associations between beverage intake and colorectal neoplasia risk. J Natl Cancer Inst. 2010 Jun 2;102(11):749-51. doi: 10.1093/jnci/djq158. Epub 2010 May 7. PMID: 20453202.
17. Kim EH, Hankinson SE, Eliassen AH, Willett WC. A prospective study of grapefruit and grapefruit juice intake and breast cancer risk. Br J Cancer. 2008 Jan 15;98(1):240-1. doi: 10.1038/sj.bjc.6604105. Epub 2007 Nov 20. PMID: 18026192; PMCID: PMC2359690.
18. Lim U, Subar AF, Mouw T, Hartge P, Morton LM, Stolzenberg-Solomon R, Campbell D, Hollenbeck AR, Schatzkin A. Consumption of aspartame-containing beverages and incidence of hematopoietic and brain malignancies. Cancer Epidemiol Biomarkers Prev. 2006 Sep;15(9):1654-9. doi: 10.1158/1055-9965.EPI-06-0203. PMID: 16985027.
19. Mayne ST, Risch HA, Dubrow R, Chow WH, Gammon MD, Vaughan TL, Borchardt L, Schoenberg JB, Stanford JL, West AB, Rotterdam H, Blot WJ, Fraumeni JF Jr. Carbonated soft drink consumption and risk of esophageal adenocarcinoma. J Natl Cancer Inst. 2006 Jan 4;98(1):72-5. doi: 10.1093/jnci/djj007. PMID: 16391374.
20. Hirvonen T, Mennen LI, de Bree A, Castetbon K, Galan P, Bertrais S, Arnault N, Hercberg S. Consumption of antioxidant-rich beverages and risk for breast cancer in French women. Ann Epidemiol. 2006 Jul;16(7):503-8. doi: 10.1016/j.annepidem.2005.09.011. Epub 2006 Jan 9. PMID: 16406814.
21. Rashidkhani B, Akesson A, Lindblad P, Wolk A. Major dietary patterns and risk of renal cell carcinoma in a prospective cohort of Swedish women. J Nutr. 2005 Jul;135(7):1757-62. doi: 10.1093/jn/135.7.1757. PMID: 15987861.
22. Slattery ML, Caan BJ, Anderson KE, Potter JD. Intake of fluids and methylxanthine-containing beverages: association with colon cancer. Int J Cancer. 1999 Apr 12;81(2):199-204. doi: 10.1002/(sici)1097-0215(19990412)81:2<199::aid-ijc6>3.0.co;2-7. PMID: 10188719.
23. Galanis DJ, Kolonel LN, Lee J, Nomura A. Intakes of selected foods and beverages and the incidence of gastric cancer among the Japanese residents of Hawaii: a prospective study. Int J Epidemiol. 1998 Apr;27(2):173-80. doi: 10.1093/ije/27.2.173. PMID: 9602395.
24. Bravo P, Del Rey Calero J, Sánchez J, Conde M. Edulcorantes artificiales como factor de riesgo del cáncer de vejiga (Artificial sweeteners as a risk factor for cancer of the bladder). Rev Sanid Hig Publica (Madr). 1987 Mar-Apr;61(3-4):301-7. Spanish. PMID: 2446378.
25. Morgan RW, Jain MG. Bladder cancer: smoking, beverages and artificial sweeteners. Can Med Assoc J. 1974 Nov 16;111(10):1067-70. PMID: 4429932; PMCID: PMC1955852.
26. Kobayashi M, Sasazuki S, Shimazu T, Sawada N, Yamaji T, Iwasaki M, Mizoue T, Tsugane S. Association of dietary diversity with total mortality and major causes of mortality in the Japanese population: JPHC study. Eur J Clin Nutr. 2020 Jan;74(1):54-66. doi: 10.1038/s41430-019-0416-y. Epub 2019 Mar 19. PMID: 30890778.
27. Ramne S, Alves Dias J, González-Padilla E, Olsson K, Lindahl B, Engström G, Ericson U, Johansson I, Sonestedt E. Association between added sugar intake and mortality is nonlinear and dependent on sugar source in 2 Swedish population-based prospective cohorts. Am J Clin Nutr. 2019 Feb 1;109(2):411-423. doi: 10.1093/ajcn/nqy268. PMID: 30590448.
28. Mytton OT, Forouhi NG, Scarborough P, Lentjes M, Luben R, Rayner M, Khaw KT, Wareham NJ, Monsivais P. Association between intake of less-healthy foods defined by the United Kingdom's nutrient profile model and cardiovascular disease: A population-based cohort study. PLoS Med. 2018 Jan 4;15(1):e1002484. doi: 10.1371/journal.pmed.1002484. PMID: 29300725; PMCID: PMC5754044.
29. Liu L, Nishihara R, Qian ZR, Tabung FK, Nevo D, Zhang X, Song M, Cao Y, Mima K, Masugi Y, Shi Y, da Silva A, Twombly T, Gu M, Li W, Hamada T, Kosumi K, Inamura K, Nowak JA, Drew DA, Lochhead P, Nosho K, Wu K, Wang M, Garrett WS, Chan AT, Fuchs CS, Giovannucci EL, Ogino S. Association Between Inflammatory Diet Pattern and Risk of Colorectal Carcinoma Subtypes Classified by Immune Responses to Tumor. Gastroenterology. 2017 Dec;153(6):1517-1530.e14. doi: 10.1053/j.gastro.2017.08.045. Epub 2017 Sep 1. PMID: 28865736; PMCID: PMC5705461.
30. Voortman T, Kiefte-de Jong JC, Ikram MA, Stricker BH, van Rooij FJA, Lahousse L, Tiemeier H, Brusselle GG, Franco OH, Schoufour JD. Adherence to the 2015 Dutch dietary guidelines and risk of non-communicable diseases and mortality in the Rotterdam Study. Eur J Epidemiol. 2017 Nov;32(11):993-1005. doi: 10.1007/s10654-017-0295-2. Epub 2017 Aug 19. PMID: 28825166; PMCID: PMC5684301.
31. Deschasaux M, Julia C, Kesse-Guyot E, Lécuyer L, Adriouch S, Méjean C, Ducrot P, Péneau S, Latino-Martel P, Fezeu LK, Fassier P, Hercberg S, Touvier M. Are self-reported unhealthy food choices associated with an increased risk of breast cancer? Prospective cohort study using the British Food Standards Agency nutrient profiling system. BMJ Open. 2017 Jun 8;7(6):e013718. doi: 10.1136/bmjopen-2016-013718. PMID: 28600360; PMCID: PMC5577898.
32. Nanri A, Mizoue T, Shimazu T, Ishihara J, Takachi R, Noda M, Iso H, Sasazuki S, Sawada N, Tsugane S; Japan Public Health Center-Based Prospective Study Group. Dietary patterns and all-cause, cancer, and cardiovascular disease mortality in Japanese men and women: The Japan public health center-based prospective study. PLoS One. 2017 Apr 26;12(4):e0174848. doi: 10.1371/journal.pone.0174848. PMID: 28445513; PMCID: PMC5405917.
33. Makarem N, Bandera EV, Lin Y, Jacques PF, Hayes RB, Parekh N. Consumption of Sugars, Sugary Foods, and Sugary Beverages in Relation to Adiposity-Related Cancer Risk in the Framingham Offspring Cohort (1991-2013). Cancer Prev Res (Phila). 2018 Jun;11(6):347-358. doi: 10.1158/1940-6207.CAPR-17-0218. Epub 2018 Apr 19. PMID: 29674390; PMCID: PMC7225083.
34. Tasevska N, Park Y, Jiao L, Hollenbeck A, Subar AF, Potischman N. Sugars and risk of mortality in the NIH-AARP Diet and Health Study. Am J Clin Nutr. 2014 May;99(5):1077-88. doi: 10.3945/ajcn.113.069369. Epub 2014 Feb 19. PMID: 24552754; PMCID: PMC3985213.
35. Scarborough P, Adhikari V, Harrington RA, Elhussein A, Briggs A, Rayner M, Adams J, Cummins S, Penney T, White M. Impact of the announcement and implementation of the UK Soft Drinks Industry Levy on sugar content, price, product size and number of available soft drinks in the UK, 2015-19: A controlled interrupted time series analysis. PLoS Med. 2020 Feb 11;17(2):e1003025. doi: 10.1371/journal.pmed.1003025. PMID: 32045418; PMCID: PMC7012398.
36. Avery KN, Donovan JL, Gilbert R, Davis M, Emmett P, Down L, Oliver S, Neal DE, Hamdy FC, Lane JA. Men with prostate cancer make positive dietary changes following diagnosis and treatment. Cancer Causes Control. 2013 Jun;24(6):1119-28. doi: 10.1007/s10552-013-0189-x. Epub 2013 Mar 22. PMID: 23519639.
37. Couto E, Sandin S, Löf M, Ursin G, Adami HO, Weiderpass E. Mediterranean dietary pattern and risk of breast cancer. PLoS One. 2013;8(2):e55374. doi: 10.1371/journal.pone.0055374. Epub 2013 Feb 4. PMID: 23390532; PMCID: PMC3563544.
38. Fung TT, Hu FB, Schulze M, Pollak M, Wu T, Fuchs CS, Giovannucci E. A dietary pattern that is associated with C-peptide and risk of colorectal cancer in women. Cancer Causes Control. 2012 Jun;23(6):959-65. doi: 10.1007/s10552-012-9969-y. Epub 2012 Apr 26. PMID: 22535146; PMCID: PMC3572718.
39. Ros MM, Bas Bueno-de-Mesquita HB, Büchner FL, Aben KK, Kampman E, Egevad L, Overvad K, Tjønneland A, Roswall N, Clavel-Chapelon F, Kaaks R, Chang-Claude J, Boeing H, Weikert S, Trichopoulou A, Orfanos P, Stasinopulou G, Saieva C, Krogh V, Vineis P, Tumino R, Mattiello A, Peeters PH, van Duijnhoven FJ, Lund E, Gram IT, Chirlaque MD, Barricarte A, Rodríguez L, Molina E, Gonzalez C, Dorronsoro M, Manjer J, Ehrnström R, Ljungberg B, Allen NE, Roddam AW, Khaw KT, Wareham N, Boffetta P, Slimani N, Michaud DS, Kiemeney LA, Riboli E. Fluid intake and the risk of urothelial cell carcinomas in the European Prospective Investigation into Cancer and Nutrition (EPIC). Int J Cancer. 2011 Jun 1;128(11):2695-708. doi: 10.1002/ijc.25592. Epub 2010 Oct 8. PMID: 20715171.
40. Zhong GC, Li QJ, Yang PF, Wang YB, Hao FB, Wang K, Hu JJ, Wu JJ. Low-carbohydrate diets and the risk of pancreatic cancer: a large prospective cohort study. Carcinogenesis. 2021 Jan 22:bgab006. doi: 10.1093/carcin/bgab006. Epub ahead of print. PMID: 33480980.
41. Simons CC, Leurs LJ, Weijenberg MP, Schouten LJ, Goldbohm RA, van den Brandt PA. Fluid intake and colorectal cancer risk in the Netherlands Cohort Study. Nutr Cancer. 2010;62(3):307-21. doi: 10.1080/01635580903407098. PMID: 20358468.
42. Cottet V, Touvier M, Fournier A, Touillaud MS, Lafay L, Clavel-Chapelon F, Boutron-Ruault MC. Postmenopausal breast cancer risk and dietary patterns in the E3N-EPIC prospective cohort study. Am J Epidemiol. 2009 Nov 15;170(10):1257-67. doi: 10.1093/aje/kwp257. Epub 2009 Oct 14. PMID: 19828509.
43. Hu J, Mao Y, DesMeules M, Csizmadi I, Friedenreich C, Mery L; Canadian Cancer Registries Epidemiology Research Group. Total fluid and specific beverage intake and risk of renal cell carcinoma in Canada. Cancer Epidemiol. 2009 Nov;33(5):355-62. doi: 10.1016/j.canep.2009.10.004. Epub 2009 Nov 7. PMID: 19896918.
44. Oba S, Nagata C, Nakamura K, Fujii K, Kawachi T, Takatsuka N, Shimizu H. Diet based on the Japanese Food Guide Spinning Top and subsequent mortality among men and women in a general Japanese population. J Am Diet Assoc. 2009 Sep;109(9):1540-7. doi: 10.1016/j.jada.2009.06.367. PMID: 19699833.
45. Schulz M, Hoffmann K, Weikert C, Nöthlings U, Schulze MB, Boeing H. Identification of a dietary pattern characterized by high-fat food choices associated with increased risk of breast cancer: the European Prospective Investigation into Cancer and Nutrition (EPIC)-Potsdam Study. Br J Nutr. 2008 Nov;100(5):942-6. doi: 10.1017/S0007114508966149. PMID: 18377685.
46. Bao Y, Stolzenberg-Solomon R, Jiao L, Silverman DT, Subar AF, Park Y, Leitzmann MF, Hollenbeck A, Schatzkin A, Michaud DS. Added sugar and sugar-sweetened foods and beverages and the risk of pancreatic cancer in the National Institutes of Health-AARP Diet and Health Study. Am J Clin Nutr. 2008 Aug;88(2):431-40. doi: 10.1093/ajcn/88.2.431. PMID: 18689380; PMCID: PMC3500146.
47. Nagel G, Zoller D, Ruf T, Rohrmann S, Linseisen J. Long-term reproducibility of a food-frequency questionnaire and dietary changes in the European Prospective Investigation into Cancer and Nutrition (EPIC)-Heidelberg cohort. Br J Nutr. 2007 Jul;98(1):194-200. doi: 10.1017/S0007114507691636. Epub 2007 Mar 19. PMID: 17367573.
48. Kesse E, Clavel-Chapelon F, Boutron-Ruault MC. Dietary patterns and risk of colorectal tumors: a cohort of French women of the National Education System (E3N). Am J Epidemiol. 2006 Dec 1;164(11):1085-93. doi: 10.1093/aje/kwj324. Epub 2006 Sep 21. PMID: 16990408; PMCID: PMC2175071.
49. Waijers PM, Ocké MC, van Rossum CT, Peeters PH, Bamia C, Chloptsios Y, van der Schouw YT, Slimani N, Bueno-de-Mesquita HB. Dietary patterns and survival in older Dutch women. Am J Clin Nutr. 2006 May;83(5):1170-6. doi: 10.1093/ajcn/83.5.1170. PMID: 16685062.
50. Lim U, Subar AF, Mouw T, Hartge P, Morton LM, Stolzenberg-Solomon R, Campbell D, Hollenbeck AR, Schatzkin A. Consumption of aspartame-containing beverages and incidence of hematopoietic and brain malignancies. Cancer Epidemiol Biomarkers Prev. 2006 Sep;15(9):1654-9. doi: 10.1158/1055-9965.EPI-06-0203. PMID: 16985027.
51. Taha Z, Eltom SE. The Role of Diet and Lifestyle in Women with Breast Cancer: An Update Review of Related Research in the Middle East. Biores Open Access. 2018 May 1;7(1):73-80. doi: 10.1089/biores.2018.0004. PMID: 29862141; PMCID: PMC5982158.
52. Braverman-Bronstein A, Camacho-García-Formentí D, Zepeda-Tello R, Cudhea F, Singh GM, Mozaffarian D, Barrientos-Gutierrez T. Mortality attributable to sugar sweetened beverages consumption in Mexico: an update. Int J Obes (Lond). 2020 Jun;44(6):1341-1349. doi: 10.1038/s41366-019-0506-x. Epub 2019 Dec 10. PMID: 31822805.
53. [J.E. Monteiro dos Santos](https://www.researchgate.net/scientific-contributions/JE-Monteiro-dos-Santos-2147857050), Dietary Patterns and Unhealthy Behaviors in Brazil: The Current Scenario. Journal of Global Oncology. 2018; 4(4_suppl_2):00-00. DOI: 10.1200/jgo.18.49700
54. Liu L, Nishihara R, Qian ZR, Tabung FK, Nevo D, Zhang X, Song M, Cao Y, Mima K, Masugi Y, Shi Y, da Silva A, Twombly T, Gu M, Li W, Hamada T, Kosumi K, Inamura K, Nowak JA, Drew DA, Lochhead P, Nosho K, Wu K, Wang M, Garrett WS, Chan AT, Fuchs CS, Giovannucci EL, Ogino S. Association Between Inflammatory Diet Pattern and Risk of Colorectal Carcinoma Subtypes Classified by Immune Responses to Tumor. Gastroenterology. 2017 Dec;153(6):1517-1530.e14. doi: 10.1053/j.gastro.2017.08.045. Epub 2017 Sep 1. PMID: 28865736; PMCID: PMC5705461.
55. Fassier P, Zelek L, Lécuyer L, Bachmann P, Touillaud M, Druesne-Pecollo N, Galan P, Cohen P, Hoarau H, Latino-Martel P, Kesse-Guyot E, Baudry J, Hercberg S, Deschasaux M, Touvier M. Modifications in dietary and alcohol intakes between before and after cancer diagnosis: Results from the prospective population-based NutriNet-Santé cohort. Int J Cancer. 2017 Aug 1;141(3):457-470. doi: 10.1002/ijc.30704. Epub 2017 May 15. PMID: 28335085; PMCID: PMC5655904.
56. Kurotani K, Honjo K, Nakaya T, Ikeda A, Mizoue T, Sawada N, Tsugane S; Japan Public Health Center-based Prospective Study Group. Diet Quality Affects the Association between Census-Based Neighborhood Deprivation and All-Cause Mortality in Japanese Men and Women: The Japan Public Health Center-Based Prospective Study. Nutrients. 2019 Sep 12;11(9):2194. doi: 10.3390/nu11092194. PMID: 31547299; PMCID: PMC6770038.
57. Sotos-Prieto M, Mattei J, Cook NR, Hu FB, Willett WC, Chiuve SE, Rimm EB, Sesso HD. Association Between a 20-Year Cardiovascular Disease Risk Score Based on Modifiable Lifestyles and Total and Cause-Specific Mortality Among US Men and Women. J Am Heart Assoc. 2018 Nov 6;7(21):e010052. doi: 10.1161/JAHA.118.010052. PMID: 30373451; PMCID: PMC6404201.
58. García-Chávez CG, Monterrubio-Flores E, Ramírez-Silva I, Aburto TC, Pedraza LS, Rivera-Dommarco J. Contribución de los alimentos a la ingesta total de energía en la dieta de los mexicanos mayores de cinco años (Food contribution to total daily energy intake in the Mexican population older than five years). Salud Publica Mex. 2020 Mar-Apr;62(2):166-180. Spanish. doi: 10.21149/10636. PMID: 32237559.
59. Schoufour JD, Kiefte-de Jong JC, Voortman T. Naleven van voedingsrichtlijnen (Adherence to the 2015 Dutch dietary guidelines and risk of ten non-communicable diseases and mortality in the Rotterdam Study). Ned Tijdschr Geneeskd. 2018 Sep 19;162:D2724. Dutch. PMID: 30306761.
60. Radkevich LA, Radkevich DA. The dietary patterns are a modifying risk factor for breast cancer: An ecological study. Dokl Biol Sci. 2017 Jan;472(1):21-27. doi: 10.1134/S0012496617010070. Epub 2017 Apr 21. PMID: 28429260.
61. Johnson RK. Reducing Intake of Sugar-Sweetened Beverages Is Vital to Improving Our Nation's Health. Circulation. 2016 Jan 26;133(4):347-9. doi: 10.1161/CIRCULATIONAHA.115.020453. Epub 2016 Jan 11. PMID: 26755504.
62. Tseng T S , Lin H Y , Griffiths L , et al. Sugar intake from sugar-sweetened beverage among cancer and non-cancer individuals: the NHANES study(J). Translational Cancer Research, 2016, 5(S5):S1019-S1028.
63. Whalen KA, Judd S, McCullough ML, Flanders WD, Hartman TJ, Bostick RM. Paleolithic and Mediterranean Diet Pattern Scores Are Inversely Associated with All-Cause and Cause-Specific Mortality in Adults. J Nutr. 2017 Apr;147(4):612-620. doi: 10.3945/jn.116.241919. Epub 2017 Feb 8. PMID: 28179490; PMCID: PMC5368578.
64. Ruff RR. Sugar-sweetened beverage consumption is linked to global adult morbidity and mortality through diabetes mellitus, cardiovascular disease and adiposity-related cancers. Evid Based Med. 2015 Dec;20(6):223-4. doi: 10.1136/ebmed-2015-110267. Epub 2015 Oct 6. PMID: 26442567.
65. Mousavi SM, Hashemi SA, Zarei M, Gholami A, Lai CW, Chiang WH, Omidifar N, Bahrani S, Mazraedoost S. Recent Progress in Chemical Composition, Production, and Pharmaceutical Effects of Kombucha Beverage: A Complementary and Alternative Medicine. Evid Based Complement Alternat Med. 2020 Nov 18;2020:4397543. doi: 10.1155/2020/4397543. PMID: 33281911; PMCID: PMC7688354.
66. Cuomo R, Andreozzi P, Zito FP. Alcoholic beverages and carbonated soft drinks: consumption and gastrointestinal cancer risks. Cancer Treat Res. 2014;159:97-120. doi: 10.1007/978-3-642-38007-5_7. PMID: 24114477.
67. Shin WK, Lee HW, Shin A, Lee JK, Lee SA, Lee JE, Kang D. Multi-Grain Rice Diet Decreases Risk of Breast Cancer in Korean Women: Results from the Health Examinees Study. Nutrients. 2020 Jul 29;12(8):2273. doi: 10.3390/nu12082273. PMID: 32751310; PMCID: PMC7468941.
68. Song YJ, Kristal AR, Wicklund KG, Cushing-Haugen KL, Rossing MA. Coffee, tea, colas, and risk of epithelial ovarian cancer. Cancer Epidemiol Biomarkers Prev. 2008 Mar;17(3):712-6. doi: 10.1158/1055-9965.EPI-07-2511. PMID: 18349292.
69. Lee JE, Hunter DJ, Spiegelman D, Adami HO, Bernstein L, van den Brandt PA, Buring JE, Cho E, English D, Folsom AR, Freudenheim JL, Gile GG, Giovannucci E, Horn-Ross PL, Leitzmann M, Marshall JR, Männistö S, McCullough ML, Miller AB, Parker AS, Pietinen P, Rodriguez C, Rohan TE, Schatzkin A, Schouten LJ, Willett WC, Wolk A, Zhang SM, Smith-Warner SA. Intakes of coffee, tea, milk, soda and juice and renal cell cancer in a pooled analysis of 13 prospective studies. Int J Cancer. 2007 Nov 15;121(10):2246-53. doi: 10.1002/ijc.22909. PMID: 17583573.
70. Mallath MK. Re: Carbonated soft drink consumption and risk of esophageal adenocarcinoma. J Natl Cancer Inst. 2006 May 3;98(9):644-5; author reply 646-7. doi: 10.1093/jnci/djj169. PMID: 16670392.
71. Chyou PH, Nomura AM, Stemmermann GN. Diet, alcohol, smoking and cancer of the upper aerodigestive tract: a prospective study among Hawaii Japanese men. Int J Cancer. 1995 Mar 3;60(5):616-21. doi: 10.1002/ijc.2910600508. PMID: 7860134.
72. Luo X, Sui J, Yang W, Sun Q, Ma Y, Simon TG, Liang G, Meyerhardt JA, Chan AT, Giovannucci EL, Zhang X. Type 2 Diabetes Prevention Diet and Hepatocellular Carcinoma Risk in US Men and Women. Am J Gastroenterol. 2019 Dec;114(12):1870-1877. doi: 10.14309/ajg.0000000000000450. PMID: 31688024; PMCID: PMC6893135.
73. Haridass V, Ziogas A, Neuhausen SL, Anton-Culver H, Odegaard AO. Diet Quality Scores Inversely Associated with Postmenopausal Breast Cancer Risk Are Not Associated with Premenopausal Breast Cancer Risk in the California Teachers Study. J Nutr. 2018 Nov 1;148(11):1830-1837. doi: 10.1093/jn/nxy187. PMID: 30247577.
74. Sharma I, Roebothan B, Zhu Y, Woodrow J, Parfrey PS, Mclaughlin JR, Wang PP. Hypothesis and data-driven dietary patterns and colorectal Cancer survival: findings from Newfoundland and Labrador colorectal Cancer cohort. Nutr J. 2018 May 25;17(1):55. doi: 10.1186/s12937-018-0362-x. PMID: 29793493; PMCID: PMC5968482.
75. Glenn BA, Hamilton AS, Nonzee NJ, Maxwell AE, Crespi CM, Ryerson AB, Chang LC, Deapen D, Bastani R. Obesity, physical activity, and dietary behaviors in an ethnically-diverse sample of cancer survivors with early onset disease. J Psychosoc Oncol. 2018 Jul-Aug;36(4):418-436. doi: 10.1080/07347332.2018.1448031. Epub 2018 May 15. PMID: 29764334; PMCID: PMC6209096.
76. Fassier P, Zelek L, Lécuyer L, Bachmann P, Touillaud M, Druesne-Pecollo N, Galan P, Cohen P, Hoarau H, Latino-Martel P, Kesse-Guyot E, Baudry J, Hercberg S, Deschasaux M, Touvier M. Modifications in dietary and alcohol intakes between before and after cancer diagnosis: Results from the prospective population-based NutriNet-Santé cohort. Int J Cancer. 2017 Aug 1;141(3):457-470. doi: 10.1002/ijc.30704. Epub 2017 May 15. PMID: 28335085; PMCID: PMC5655904.
77. Fung TT, Kashambwa R, Sato K, Chiuve SE, Fuchs CS, Wu K, Giovannucci E, Ogino S, Hu FB, Meyerhardt JA. Post diagnosis diet quality and colorectal cancer survival in women. PLoS One. 2014 Dec 15;9(12):e115377. doi: 10.1371/journal.pone.0115377. PMID: 25506700; PMCID: PMC4266679.
78. Fuchs MA, Sato K, Niedzwiecki D, Ye X, Saltz LB, Mayer RJ, Mowat RB, Whittom R, Hantel A, Benson A, Atienza D, Messino M, Kindler H, Venook A, Ogino S, Wu K, Willett WC, Giovannucci EL, Meyerhardt JA. Sugar-sweetened beverage intake and cancer recurrence and survival in CALGB 89803 (Alliance). PLoS One. 2014 Jun 17;9(6):e99816. doi: 10.1371/journal.pone.0099816. PMID: 24937507; PMCID: PMC4061031.
79. Song YJ, Kristal AR, Wicklund KG, Cushing-Haugen KL, Rossing MA. Coffee, tea, colas, and risk of epithelial ovarian cancer. Cancer Epidemiol Biomarkers Prev. 2008 Mar;17(3):712-6. doi: 10.1158/1055-9965.EPI-07-2511. PMID: 18349292.
80. Ruff, R. R. Sugar-sweetened beverage consumption is linked to global adult morbidity and mortality through diabetes mellitus, cardiovascular disease and adiposity-related cancers. Evidence-Based Medicine. 2015; 6 (20): 223-224
81. Martínez-González MA, Ruiz-Canela M. Preventing heart failure: sweetened beverages and healthy lifestyles. Heart. 2015;101(24): 1935‐1937.
82. Kumar GS, Pan L, Park S, et al. Sugar-sweetened beverage consumption among adults -- 18 states, 2012. MMWR Morb Mortal Wkly Rep. 2014;63(32):686‐690.
83. Gardener H, Rundek T, Wright CB, Elkind MS, Sacco RL. Coffee and tea consumption are inversely associated with mortality in a multiethnic urban population. J Nutr. 2013;143(8):1299‐1308.
84. Rahman I, Wolk A, Larsson SC. The relationship between sweetened beverage consumption and risk of heart failure in men. Heart. 2015;101(24):1961‐1965.
85. Razvodovsky YE. The effects of beverage type on pancreatitis mortality rate in Russia. Pancreas. 2015;44(5):832‐833.
86. Goh GB, Chow WC, Wang R, Yuan JM, Koh WP. Coffee, alcohol and other beverages in relation to cirrhosis mortality: the Singapore Chinese Health Study. Hepatology. 2014;60(2):661‐669.
87. Freedman ND, Park Y, Abnet CC, Hollenbeck AR, Sinha R. Association of coffee drinking with total and cause-specific mortality (published correction appears in N Engl J Med. 2012 Jul 19;367(3):285). N Engl J Med. 2012;366(20):1891‐1904.
88. Rivera-Paredez B, Muñoz-Aguirre P, Torres-Ibarra L, et al. Patterns of beverage consumption and risk of CHD among Mexican adults. Br J Nutr. 2018;120(2):210‐219.
89. van Dongen LH, Mölenberg FJ, Soedamah-Muthu SS, Kromhout D, Geleijnse JM. Coffee consumption after myocardial infarction and risk of cardiovascular mortality: a prospective analysis in the Alpha Omega Cohort. Am J Clin Nutr. 2017;106(4):1113‐1120.
90. Mytton OT, Forouhi NG, Scarborough P, et al. Association between intake of less-healthy foods defined by the United Kingdom's nutrient profile model and cardiovascular disease: A population-based cohort study. PLoS Med. 2018;15(1):e1002484. Published 2018 Jan 4.
91. Hu Y, Ding M, Yuan C, et al. Association Between Coffee Intake After Diagnosis of Colorectal Cancer and Reduced Mortality. Gastroenterology. 2018;154(4):916‐926.e9.
92. Collin LJ, Judd S, Safford M, Vaccarino V, Welsh JA. Association of Sugary Beverage Consumption With Mortality Risk in US Adults: A Secondary Analysis of Data From the REGARDS Study. JAMA Netw Open. 2019;2(5):e193121. Published 2019 May 3.
93. Guercio BJ, Zhang S, Niedzwiecki D, et al. Associations of artificially sweetened beverage intake with disease recurrence and mortality in stage III colon cancer: Results from CALGB 89803 (Alliance). PLoS One. 2018;13(7):e0199244. Published 2018 Jul 19.
94. Asadi Z, Shafiee M, Sadabadi F, Heidari-Bakavoli A, Moohebati M, Khorrami MS, Darroudi S, Heidari S, Hoori T, Tayefi M, Mohammadi F, Esmaeily H, Safarian M, Ghayour-Mobarhan M, Ferns GA. Association of dietary patterns and risk of cardiovascular disease events in the MASHAD cohort study. J Hum Nutr Diet. 2019 Dec;32(6):789-801.
95. Gómez-Donoso C, Martínez-González MÁ, Perez-Cornago A, Sayón-Orea C, Martínez JA, Bes-Rastrollo M. Association between the nutrient profile system underpinning the Nutri-Score front-of-pack nutrition label and mortality in the SUN project: A prospective cohort study. Clin Nutr. 2020 Jul 17:S0261-5614(20)30359-9.
96. Rezende LF, Azeredo CM, Canella DS, Luiz Odo C, Levy RB, Eluf-Neto J. Coronary heart disease mortality, cardiovascular disease mortality and all-cause mortality attributable to dietary intake over 20years in Brazil. Int J Cardiol. 2016;217:64‐68
97. Goh GB, Chow WC, Wang R, Yuan JM, Koh WP. Coffee, alcohol and other beverages in relation to cirrhosis mortality: the Singapore Chinese Health Study. Hepatology. 2014;60(2):661‐669.
98. Gardener H, Rundek T, Wright CB, Elkind MS, Sacco RL. Coffee and tea consumption are inversely associated with mortality in a multiethnic urban population. J Nutr. 2013;143(8):1299‐1308
99. Akinyemiju T, Moore JX, Pisu M, et al. A prospective study of dietary patterns and cancer mortality among Blacks and Whites in the REGARDS cohort. Int J Cancer. 2016;139(10):2221‐2231.

# Appendix 2 the lists of included studies

1. McCullough ML, Teras LR, Shah R, et al. Artificially and sugar-sweetened carbonated beverage consumption is not associated with risk of lymphoid neoplasms in older men and women. J Nutr. 2014; 144(12):2041-9.

2. Inoue-Choi M, Robien K, Mariani A, et al. Sugar-sweetened beverage intake and the risk of type I and type II endometrial cancer among postmenopausal women. Cancer Epidemiol Biomarkers Prev. 2013; 22(12):2384-94.

3. Drake I, Sonestedt E, Gullberg B, et al. Dietary intakes of carbohydrates in relation to prostate cancer risk: a prospective study in the Malmö Diet and Cancer cohort. Am J Clin Nutr. 2012; 96(6):1409-18.

4. Ren JS, Freedman ND, Kamangar F, et al. Tea, coffee, carbonated soft drinks and upper gastrointestinal tract cancer risk in a large United States prospective cohort study. Eur J Cancer. 2010; 46(10):1873-81.

5. Mueller NT, Odegaard A, Anderson K, et al. Soft drink and juice consumption and risk of pancreatic cancer: the Singapore Chinese Health Study. Cancer Epidemiol Biomarkers Prev. 2010; 19(2):447-55.

6. Nöthlings U, Murphy SP, Wilkens LR, et al. Dietary glycemic load, added sugars, and carbohydrates as risk factors for pancreatic cancer: the Multiethnic Cohort Study. Am J Clin Nutr. 2007; 86(5):1495-501.

7. Larsson SC, Bergkvist L, Wolk A. Consumption of sugar and sugar-sweetened foods and the risk of pancreatic cancer in a prospective study. Am J Clin Nutr. 2006 Nov;84(5):1171-6.

8. Lee JE, Giovannucci E, Smith-Warner SA, et al. Total fluid intake and use of individual beverages and risk of renal cell cancer in two large cohorts. Cancer Epidemiol Biomarkers Prev. 2006;15(6):1204-11.

9. Bassett JK, Milne RL, English DR, et al. Consumption of sugar-sweetened and artificially sweetened soft drinks and risk of cancers not related to obesity. Int J Cancer. 2020;146(12):3329-3334. doi: 10.1002/ijc.32772

10. Luo X, Sui J, Yang W, et al. Type 2 Diabetes Prevention Diet and Hepatocellular Carcinoma Risk in US Men and Women. Am J Gastroenterol. 2019 Dec;114(12):1870-1877.

11. Pacheco LS, Anderson CAM, Lacey JV Jr, et al. Sugar-sweetened beverages and colorectal cancer risk in the California Teachers Study. PLoS One. 2019; 14(10):e0223638.

12. Miles FL, Neuhouser ML, Zhang ZF. Concentrated sugars and incidence of prostate cancer in a prospective cohort. Br J Nutr. 2018; 120(6):703-710.

13. Zamora-Ros R, Béraud V, Franceschi S, et al. Consumption of fruits, vegetables and fruit juices and differentiated thyroid carcinoma risk in the European Prospective Investigation into Cancer and Nutrition (EPIC) study. Int J Cancer. 2018; 142(3):449-459.

14. Larsson SC, Giovannucci EL, Wolk A. Sweetened Beverage Consumption and Risk of Biliary Tract and Gallbladder Cancer in a Prospective Study. J Natl Cancer Inst. 2016; 108(10): djw125.

15. Ellison LF. Tea and other beverage consumption and prostate cancer risk: a Canadian retrospective cohort study. Eur J Cancer Prev. 2000; 9(2):125-30.

16. Debras C, Chazelas E, Srour B, et al. Total and added sugar intakes, sugar types, and cancer risk: results from the prospective NutriNet-Santé cohort. Am J Clin Nutr. 2020; 112(5):1267-1279.

17. Makarem N, Bandera EV, Lin Y, et al. Consumption of Sugars, Sugary Foods, and Sugary Beverages in Relation to Adiposity-Related Cancer Risk in the Framingham Offspring Cohort (1991-2013). Cancer Prev Res (Phila). 2018 Jun;11(6):347-358.

18. Fung TT, Hu FB, Wu K, et al. The Mediterranean and Dietary Approaches to Stop Hypertension (DASH) diets and colorectal cancer. Am J Clin Nutr. 2010;92(6):1429-35.

19. Bao Y, Stolzenberg-Solomon R, Jiao L, et al. Added sugar and sugar-sweetened foods and beverages and the risk of pancreatic cancer in the National Institutes of Health-AARP Diet and Health Study. Am J Clin Nutr. 2008; 88(2):431-40.

20. Michaud DS, Spiegelman D, Clinton SK, et al. Fluid intake and the risk of bladder cancer in men. N Engl J Med. 1999; 340(18):1390-7.

21. Romanos-Nanclares A, Toledo E, Gardeazabal I, et al. Sugar-sweetened beverage consumption and incidence of breast cancer: the Seguimiento Universidad de Navarra (SUN) Project. Eur J Nutr. 2019; 58(7):2875-2886.

22. Stepien M, Duarte-Salles T, Fedirko V, et al. Consumption of soft drinks and juices and risk of liver and biliary tract cancers in a European cohort. Eur J Nutr. 2016; 55(1):7-20.

23. Wu S, Cho E, Feskanich D, et al. Citrus consumption and risk of basal cell carcinoma and squamous cell carcinoma of the skin. Carcinogenesis. 2015 Oct;36(10):1162-8.

24. Wu S, Han J, Feskanich D, Cho E, et al. Citrus Consumption and Risk of Cutaneous Malignant Melanoma. J Clin Oncol. 2015; 33(23):2500-8.

25. Ros MM, Bas Bueno-de-Mesquita HB, Büchner FL, et al. Fluid intake and the risk of urothelial cell carcinomas in the European Prospective Investigation into Cancer and Nutrition (EPIC). Int J Cancer. 2011;128(11):2695-708.

26. Allen NE, Balkwill A, Beral V, et al. Fluid intake and incidence of renal cell carcinoma in UK women. Br J Cancer. 2011; 104(9):1487-92.

27. Friberg E, Wallin A, Wolk A. Sucrose, high-sugar foods, and risk of endometrial cancer--a population-based cohort study. Cancer Epidemiol Biomarkers Prev. 2011 Sep;20(9):1831-7.

28. Khan MM, Goto R, Kobayashi K, et al. Dietary habits and cancer mortality among middle aged and older Japanese living in hokkaido, Japan by cancer site and sex. Asian Pac J Cancer Prev. 2004 J; 5(1):58-65.

29. Zhang X, Albanes D, Beeson WL, et al. Risk of colon cancer and coffee, tea, and sugar-sweetened soft drink intake: pooled analysis of prospective cohort studies. J Natl Cancer Inst. 2010; 102(11):771-83.

30. Heath AK, Clasen JL, Jayanth NP, et al. Soft Drink and Juice Consumption and Renal Cell Carcinoma Incidence and Mortality in the European Prospective Investigation into Cancer and Nutrition. Cancer Epidemiol Biomarkers Prev. 202;30(6):1270-1274.

31. Arthur RS, Kirsh VA, Mossavar-Rahmani Y, et al. Sugar-containing beverages and their association with risk of breast, endometrial, ovarian and colorectal cancers among Canadian women. Cancer Epidemiol. 202; 70: 101855.

32. Yuan C, Joh HK, Wang QL, et al. Sugar-sweetened beverage and sugar consumption and colorectal cancer incidence and mortality according to anatomic subsite. Am J Clin Nutr. 2022;115(6):1481-1489.

33. Hur J, Otegbeye E, Joh HK, et al. Sugar-sweetened beverage intake in adulthood and adolescence and risk of early-onset colorectal cancer among women. Gut. 2021;70(12):2330-2336.

34. Chen CH, Tsai MK, Lee JH, et al. "Sugar-Sweetened Beverages" Is an Independent Risk From Pancreatic Cancer: Based on Half a Million Asian Cohort Followed for 25 Years. Front Oncol. 2022; 12: 835901.

35. Romanos-Nanclares A, Collins LC, Hu FB, et al. Sugar-Sweetened Beverages, Artificially Sweetened Beverages, and Breast Cancer Risk: Results From 2 Prospective US Cohorts. J Nutr. 2021; 151(9):2768-2779.

36. N. Ringel, K. Hovey, C. Andrews, et al. Association between artificially sweetened

beverages and urinary tract cancers. American Journal of Obstetrics & Gynecology. 2022; Supplement to MARCH

37. Chazelas E, Srour B, Desmetz E, et al. Sugary drink consumption and risk of cancer: results from NutriNet-Santé prospective cohort. BMJ 2019;366:l2408.

38. Odegaard AO, Koh WP, Yuan JM, Pereira MA. Beverage habits and mortality in Chinese adults. J Nutr 2015;145(3):595-604.

39. Hodge AM, Bassett JK, Milne RL, et al. Consumption of sugar-sweetened and artificially sweetened soft drinks and risk of obesity-related cancers. Public Health Nutr 2018;21(9):1618-1626.

40. Schernhammer ES, Hu FB, Giovannucci E, et al. Sugar-sweetened soft drink consumption and risk of pancreatic cancer in two prospective cohorts. Cancer Epidemiol Biomarkers Prev 2005;14(9):2098-105.

41. Navarrete-Muñoz EM, Wark PA, Romaguera D, et al. Sweet-beverage consumption and risk of pancreatic cancer in the European Prospective Investigation into Cancer and Nutrition (EPIC). Am J Clin Nutr 2016;104(3):760-8.

42. Schernhammer ES, Bertrand KA, Birmann BM, et al. Consumption of artificial sweetener- and sugar-containing soda and risk of lymphoma and leukemia in men and women. Am J Clin Nutr 2012;96(6):1419-28.

# Appendix 3 the PRISMA checklist

| **Section and Topic** | **Item #** | **Checklist item** | **Location where item is reported** |
| --- | --- | --- | --- |
| **TITLE** | | |  |
| Title | 1 | Identify the report as a systematic review. | 1 |
| **ABSTRACT** | | |  |
| Abstract | 2 | See the PRISMA 2020 for Abstracts checklist. | 2-3 |
| **INTRODUCTION** | | |  |
| Rationale | 3 | Describe the rationale for the review in the context of existing knowledge. | 4 |
| Objectives | 4 | Provide an explicit statement of the objective(s) or question(s) the review addresses. | 5 |
| **METHODS** | | |  |
| Eligibility criteria | 5 | Specify the inclusion and exclusion criteria for the review and how studies were grouped for the syntheses. | 6 |
| Information sources | 6 | Specify all databases, registers, websites, organisations, reference lists and other sources searched or consulted to identify studies. Specify the date when each source was last searched or consulted. | 5 |
| Search strategy | 7 | Present the full search strategies for all databases, registers and websites, including any filters and limits used. | 5 |
| Selection process | 8 | Specify the methods used to decide whether a study met the inclusion criteria of the review, including how many reviewers screened each record and each report retrieved, whether they worked independently, and if applicable, details of automation tools used in the process. | 7 |
| Data collection process | 9 | Specify the methods used to collect data from reports, including how many reviewers collected data from each report, whether they worked independently, any processes for obtaining or confirming data from study investigators, and if applicable, details of automation tools used in the process. | 7 |
| Data items | 10a | List and define all outcomes for which data were sought. Specify whether all results that were compatible with each outcome domain in each study were sought (e.g. for all measures, time points, analyses), and if not, the methods used to decide which results to collect. | 7 |
|  | 10b | List and define all other variables for which data were sought (e.g. participant and intervention characteristics, funding sources). Describe any assumptions made about any missing or unclear information. | 7 |
| Study risk of bias assessment | 11 | Specify the methods used to assess risk of bias in the included studies, including details of the tool(s) used, how many reviewers assessed each study and whether they worked independently, and if applicable, details of automation tools used in the process. | 7 |
| Effect measures | 12 | Specify for each outcome the effect measure(s) (e.g. risk ratio, mean difference) used in the synthesis or presentation of results. | 8 |
| Synthesis methods | 13a | Describe the processes used to decide which studies were eligible for each synthesis (e.g. tabulating the study intervention characteristics and comparing against the planned groups for each synthesis (item #5)). | 8 |
|  | 13b | Describe any methods required to prepare the data for presentation or synthesis, such as handling of missing summary statistics, or data conversions. | 8 |
|  | 13c | Describe any methods used to tabulate or visually display results of individual studies and syntheses. | 8 |
|  | 13d | Describe any methods used to synthesize results and provide a rationale for the choice(s). If meta-analysis was performed, describe the model(s), method(s) to identify the presence and extent of statistical heterogeneity, and software package(s) used. | 8 |
|  | 13e | Describe any methods used to explore possible causes of heterogeneity among study results (e.g. subgroup analysis, meta-regression). | 8 |
|  | 13f | Describe any sensitivity analyses conducted to assess robustness of the synthesized results. | 8 |
| Reporting bias assessment | 14 | Describe any methods used to assess risk of bias due to missing results in a synthesis (arising from reporting biases). | 9 |
| Certainty assessment | 15 | Describe any methods used to assess certainty (or confidence) in the body of evidence for an outcome. | 7 |
| **RESULTS** | | |  |
| Study selection | 16a | Describe the results of the search and selection process, from the number of records identified in the search to the number of studies included in the review, ideally using a flow diagram. | 9 |
|  | 16b | Cite studies that might appear to meet the inclusion criteria, but which were excluded, and explain why they were excluded. | 10 |
| Study characteristics | 17 | Cite each included study and present its characteristics. | 10 |
| Risk of bias in studies | 18 | Present assessments of risk of bias for each included study. | 10 |
| Results of individual studies | 19 | For all outcomes, present, for each study: (a) summary statistics for each group (where appropriate) and (b) an effect estimate and its precision (e.g. confidence/credible interval), ideally using structured tables or plots. | 10 |
| Results of syntheses | 20a | For each synthesis, briefly summarise the characteristics and risk of bias among contributing studies. | 11-14 |
|  | 20b | Present results of all statistical syntheses conducted. If meta-analysis was done, present for each the summary estimate and its precision (e.g. confidence/credible interval) and measures of statistical heterogeneity. If comparing groups, describe the direction of the effect. | 11-14 |
|  | 20c | Present results of all investigations of possible causes of heterogeneity among study results. | 11-14 |
|  | 20d | Present results of all sensitivity analyses conducted to assess the robustness of the synthesized results. | 11-14 |
| Reporting biases | 21 | Present assessments of risk of bias due to missing results (arising from reporting biases) for each synthesis assessed. | 11-14 |
| Certainty of evidence | 22 | Present assessments of certainty (or confidence) in the body of evidence for each outcome assessed. | 11-14 |
| **DISCUSSION** | | |  |
| Discussion | 23a | Provide a general interpretation of the results in the context of other evidence. | 14 |
|  | 23b | Discuss any limitations of the evidence included in the review. | 16-18 |
|  | 23c | Discuss any limitations of the review processes used. | 16-18 |
|  | 23d | Discuss implications of the results for practice, policy, and future research. | 18-19 |
| **OTHER INFORMATION** | | |  |
| Registration and protocol | 24a | Provide registration information for the review, including register name and registration number, or state that the review was not registered. | 5 |
|  | 24b | Indicate where the review protocol can be accessed, or state that a protocol was not prepared. | 5 |
|  | 24c | Describe and explain any amendments to information provided at registration or in the protocol. | NA |
| Support | 25 | Describe sources of financial or non-financial support for the review, and the role of the funders or sponsors in the review. | 21 |
| Competing interests | 26 | Declare any competing interests of review authors. | 21 |
| Availability of data, code and other materials | 27 | Report which of the following are publicly available and where they can be found: template data collection forms; data extracted from included studies; data used for all analyses; analytic code; any other materials used in the review. | 21 |
